# Time Rescaling of a Primal-Dual Dynamical System with Asymptotically Vanishing Damping

**DOI:** 10.1007/s00245-023-09999-9

**Published:** 2023-05-31

**Authors:** David Alexander Hulett, Dang-Khoa Nguyen

**Affiliations:** Faculty of Mathematics, University of Vienna, Oskar-Morgenstern-Platz 1, 1090 Vienna, Austria

**Keywords:** Augmented Lagrangian method, Primal-dual dynamical system, Damped inertial dynamics, Nesterov’s accelerated gradient method, Lyapunov analysis, Time rescaling, Convergence rate, Trajectory convergence, 37N40, 46N10, 65K10, 90C25

## Abstract

In this work, we approach the minimization of a continuously differentiable convex function under linear equality constraints by a second-order dynamical system with an asymptotically vanishing damping term. The system under consideration is a time rescaled version of another system previously found in the literature. We show fast convergence of the primal-dual gap, the feasibility measure, and the objective function value along the generated trajectories. These convergence rates now depend on the rescaling parameter, and thus can be improved by choosing said parameter appropriately. When the objective function has a Lipschitz continuous gradient, we show that the primal-dual trajectory asymptotically converges weakly to a primal-dual optimal solution to the underlying minimization problem. We also exhibit improved rates of convergence of the gradient along the primal trajectories and of the adjoint of the corresponding linear operator along the dual trajectories. We illustrate the theoretical outcomes and also carry out a comparison with other classes of dynamical systems through numerical experiments.

## Introduction

### Problem Statement and Motivation

In this paper we will consider the optimization problem

1.1 $$\begin{aligned} \begin{array}{ll} \min &{} f \left( x \right) , \\ \text {subject to} &{} Ax = b \end{array} \end{aligned}$$

where

1.2 $$\begin{aligned} {\left\{ \begin{array}{ll} \mathcal {X}, \mathcal {Y}\text { are real Hilbert spaces}; \\ f :\mathcal {X}\rightarrow \mathbb {R}\text { is a continuously differentiable convex function}; \\ A :\mathcal {X}\rightarrow \mathcal {Y}\text { is a continuous linear operator and } b \in \mathcal {Y}; \\ \text {the set } \mathbb {S} \text { of primal-dual optimal solutions of } (1.1) \text { is assumed to be nonempty}. \end{array}\right. } \end{aligned}$$

This model formulation underlies many important applications in various areas, such as image recovery [25], machine learning [20, 31], the energy dispatch of power grids [42, 43], distributed optimization [32, 44] and network optimization [40, 45].

In recent years, there has been a flurry of research on the relationship between continuous time dynamical systems and the numerical algorithms that arise from their discretizations. For the unconstrained optimization problem, it has been known that inertial systems with damped velocities enjoy good convergence properties. For a convex, smooth function $f: \mathcal {X} \rightarrow \mathbb {R}$, Polyak is the first to consider the *heavy ball with friction* (HBF) dynamics [37, 38]

HBF $$\begin{aligned} \ddot{x}(t) + \gamma \dot{x}(t) + \nabla f(x(t)) = 0 . \end{aligned}$$

Alvarez and Attouch continue the line of this study, focusing on inertial dynamics with a fixed viscous damping coefficient [2–4]. Later on, Cabot et al. [21, 22] consider the system that replaces $\gamma$ with a time dependent damping coefficient $\gamma \left( t \right)$. In [41], Su, Boyd, and Candès showed that it turns out one can achieve fast convergence rates by introducing a time dependent damping coefficient which vanishes in a controlled manner, neither too fast nor too slowly, as *t* goes to infinity

AVD $$\begin{aligned} \ddot{x}(t) + \frac{\alpha }{t}\dot{x}(t) + \nabla f(x(t)) = 0. \end{aligned}$$

For $\alpha \geqslant 3$, the authors showed that a solution $x :\left[ t_{0}, +\infty \right) \rightarrow \mathcal {X}$ to (AVD) satisfies $f(x(t)) - f(x_{*}) = \mathcal {O}\left( \frac{1}{t^{2}}\right)$ as $t \rightarrow +\infty$. In fact, the choice $\alpha = 3$ provides a continuous limit counterpart to Nesterov’s celebrated accelerated gradient algorithm [15, 34, 35]. Weak convergence of the trajectories to minimizers of *f* when $\alpha > 3$ has been shown by Attouch et al. in [6] and May in [33], together with the improved rates of convergence $f(x(t)) - f(x_{*}) = o\left( \frac{1}{t^{2}}\right)$ as $t\rightarrow +\infty$. In the meantime, similar results for the discrete counterpart were also reported by Chambolle and Dossal in [23], and by Attouch and Peypouquet in [13].

In [7], Attouch, Chbani, and Riahi proposed an inertial proximal type algorithm, which results from a discretization of the time rescaled (AVD) system

$$\begin{aligned} \ddot{x}(t) + \frac{\alpha }{t}\dot{x}(t) + \delta (t) \nabla f(x(t)) = 0, \end{aligned}$$

where $\delta :\left[ t_{0}, + \infty \right) \rightarrow \mathbb {R}_{+}$ is a time scaling function satisfying a certain growth condition, which enters the convergence statement by way of $f \left( x \left( t \right) \right) - f \left( x_{*} \right) = \mathcal {O}\left( \frac{1}{t^{2} \delta \left( t \right) } \right)$ as $t \rightarrow + \infty$. The resulting algorithm obtained by the authors is considerably simpler than the founding proximal point algorithm proposed by Güler in [26], while providing comparable convergence rates for the functional values.

In order to approach constrained optimization problems, Augmented Lagrangian Method (ALM) [39] (for linearly constrained problems) and Alternating Direction Method of Multipliers (ADMM) [20, 24] (for problems with separable objectives and block variables linearly coupled in the constraints) and some of their variants have been shown to enjoy substantial success. Continuous-time approaches for structured convex minimization problems formulated in the spirit of the full splitting paradigm have been recently addressed in [18] and, closely connected to our approach, in [10, 17, 27, 45], to which we will have a closer look in Subsection 2.2. The temporal discretization resulting from these dynamics gives rise to the numerical algorithm with fast convergence rates [28, 29] and with a convergence guarantee for the generated iterate [19], without additional assumptions such as strong convexity.

In this paper, we will investigate a second-order dynamical system with asymptotic vanishing damping and time rescaling term, which is associated with the optimization problem (1.1) and formulated in terms of its augmented Lagrangian. The case when the time rescaling term does not appear has been established in [17]. We show that by introducing this time rescaling function, we are able to derive faster convergence rates for the primal-dual gap, the feasibility measure, and the objective function value along the generated trajectories while still maintaining the asymptotic behaviour of the trajectories towards a primal-dual optimal solution. On the other hand, this work can also be viewed as an extension of the time rescaling technique derived in [7, 9] for the constrained case. To our knowledge, the trajectory convergence for dynamics with time scaling seems to be new in the constrained case.

### Notations and a Preliminary Result

For both Hilbert spaces $\mathcal {X}$ and $\mathcal {Y}$, the Euclidean inner product and the associated norm will be denoted by $\left\langle \cdot , \cdot \right\rangle$ and $\left\Vert \cdot \right\Vert$, respectively. The Cartesian product $\mathcal {X}\times \mathcal {Y}$ will be endowed with the inner product and the associated norm defined for $\left( x, \lambda \right) , \left( z, \mu \right) \in \mathcal {X}\times \mathcal {Y}$ as

$$\begin{aligned} \left\langle \left( x, \lambda \right) , \left( z, \mu \right) \right\rangle = \left\langle x, z \right\rangle + \left\langle \lambda , \mu \right\rangle \qquad \text { and } \qquad \left\Vert \left( x, \lambda \right) \right\Vert = \sqrt{\left\Vert x \right\Vert ^{2} + \left\Vert \lambda \right\Vert ^{2}}, \end{aligned}$$

respectively.

Let $f :\mathcal {X}\rightarrow \mathbb {R}$ be a continuously differentiable convex function such that $\nabla f$ is $\ell -$Lipschitz continuous. For every $x, y \in \mathcal {X}$ it holds (see [35, Theorem 2.1.5])

1.3 $$\begin{aligned} 0 \leqslant \dfrac{1}{2 \ell } \left\Vert \nabla f \left( x \right) - \nabla f \left( y \right) \right\Vert ^{2} \leqslant f \left( x \right) - f \left( y \right) - \left\langle \nabla f \left( y \right) , x - y \right\rangle \leqslant \dfrac{\ell }{2} \left\Vert x - y \right\Vert ^{2} . \end{aligned}$$

## The Primal-Dual Dynamical Approach

### Augmented Lagrangian Formulation

Consider the saddle point problem

2.1 $$\begin{aligned} \min _{x \in \mathcal {X}} \max _{\lambda \in \mathcal {Y}} \mathcal {L}\left( x, \lambda \right) \end{aligned}$$

associated to problem (1.1), where $\mathcal {L}:\mathcal {X}\times \mathcal {Y}\rightarrow \mathbb {R}$ denotes the *Lagrangian* function

$$\begin{aligned} \mathcal {L}\left( x, \lambda \right) := f \left( x \right) + \left\langle \lambda , Ax - b \right\rangle . \end{aligned}$$

Under the assumptions (1.2), $\mathcal {L}$ is convex with respect to $x \in \mathcal {X}$ and affine with respect to $\lambda \in \mathcal {Y}$. A pair $\left( x_{*}, \lambda _{*} \right) \in \mathcal {X}\times \mathcal {Y}$ is said to be a *saddle point* of the Lagrangian function $\mathcal {L}$ if for every $\left( x, \lambda \right) \in \mathcal {X}\times \mathcal {Y}$

2.2 $$\begin{aligned} \mathcal {L}\left( x_{*}, \lambda \right) \leqslant \mathcal {L}\left( x_{*}, \lambda _{*} \right) \leqslant \mathcal {L}\left( x, \lambda _{*} \right) . \end{aligned}$$

If $\left( x_{*}, \lambda _{*} \right) \in \mathcal {X}\times \mathcal {Y}$ is a saddle point of $\mathcal {L}$ then $x_{*} \in \mathcal {X}$ is an optimal solution of (1.1), and $\lambda _{*} \in \mathcal {Y}$ is an optimal solution of its Lagrange dual problem. If $x_{*} \in \mathcal {X}$ is an optimal solution of (1.1) and a suitable constraint qualification is fulfilled, then there exists an optimal solution $\lambda _{*} \in \mathcal {Y}$ of the Lagrange dual problem such that $\left( x_{*}, \lambda _{*} \right) \in \mathcal {X}\times \mathcal {Y}$ is a saddle point of $\mathcal {L}$. For details and insights into the topic of constraint qualifications for convex duality we refer to [14, 16].

The set of saddle points of $\mathcal {L}$, called also primal-dual optimal solutions of (1.1), will be denoted by $\mathbb {S}$ and, as stated in the assumptions, it will be assumed to be nonempty. The set of feasible points of (1.1) will be denoted by $\mathbb {F}:= \left\{ x \in \mathcal {X}:Ax = b \right\}$ and the optimal objective value of (1.1) by $f_{*}$.

The system of primal-dual optimality conditions for (1.1) reads

2.3 $$\begin{aligned} \left( x_{*}, \lambda _{*} \right) \in \mathbb {S}\Leftrightarrow {\left\{ \begin{array}{ll} \nabla _{x} \mathcal {L}\left( x_{*}, \lambda _{*} \right) &{} = 0 \\ \nabla _{\lambda } \mathcal {L}\left( x_{*}, \lambda _{*} \right) &{} = 0 \end{array}\right. } \Leftrightarrow {\left\{ \begin{array}{ll} \nabla f \left( x_{*} \right) + A^{*} \lambda _{*} &{} = 0 \\ Ax_{*} - b &{} = 0 \end{array}\right. }, \end{aligned}$$

where $A^{*}: \mathcal {Y}\rightarrow \mathcal {X}$ denotes the adjoint operator of *A*.

For $\beta \geqslant 0$, we consider also the augmented Lagrangian $\mathcal {L}_{\beta }:\mathcal {X}\times \mathcal {Y}\rightarrow \mathbb {R}$ associated with (1.1)

2.4 $$\begin{aligned} \mathcal {L}_{\beta }\left( x, \lambda \right) := \mathcal {L}\left( x, \lambda \right) + \dfrac{\beta }{2} \left\Vert Ax - b \right\Vert ^{2} = f \left( x \right) + \left\langle \lambda , Ax - b \right\rangle + \dfrac{\beta }{2} \left\Vert Ax - b \right\Vert ^{2} . \end{aligned}$$

For every $(x, \lambda ) \in \mathbb {F}\times \mathcal {Y}$ it holds

2.5 $$\begin{aligned} f \left( x \right) = \mathcal {L}_{\beta }\left( x, \lambda \right) = \mathcal {L}\left( x, \lambda \right) . \end{aligned}$$

If $\left( x_{*}, \lambda _{*} \right) \in \mathbb {S}$, then we have for every $\left( x, \lambda \right) \in \mathcal {X}\times \mathcal {Y}$

$$\begin{aligned} \mathcal {L}\left( x_{*}, \lambda \right) = \mathcal {L}_{\beta }\left( x_{*}, \lambda \right) \leqslant \mathcal {L}\left( x_{*}, \lambda _{*} \right) = \mathcal {L}_{\beta }\left( x_{*}, \lambda _{*} \right) \leqslant \mathcal {L}\left( x, \lambda _{*} \right) \leqslant \mathcal {L}_{\beta }\left( x, \lambda _{*} \right) . \end{aligned}$$

In addition, from (2.3) we have

$$\begin{aligned} \begin{aligned} \left( x_{*}, \lambda _{*} \right) \in \mathbb {S}&\Leftrightarrow {\left\{ \begin{array}{ll} \nabla f \left( x_{*} \right) + A^{*} \lambda _{*} &{} = 0 \\ Ax_{*} - b &{} = 0 \end{array}\right. } \,\Leftrightarrow \, {\left\{ \begin{array}{ll} \nabla f \left( x_{*} \right) + A^{*} \lambda _{*} + \beta A^{*}(Ax_{*} - b) &{}= 0 \\ Ax_{*} - b &{}= 0 \end{array}\right. } \\&\Leftrightarrow {\left\{ \begin{array}{ll} \nabla _{x} \mathcal {L}_{\beta }(x_{*}, \lambda _{*}) &{}= 0 \\ \nabla _{\lambda } \mathcal {L}_{\beta }(x_{*}, \lambda _{*}) &{}= 0. \end{array}\right. } \end{aligned} \end{aligned}$$

In other words, for any $\beta \geqslant 0$ the sets of saddle points of $\mathcal {L}$ and $\mathcal {L}_{\beta }$ are identical.

### The Primal-Dual Asymptotic Vanishing Damping Dynamical System with Time Rescaling

In this subsection we present the system under study, and we include a brief discussion regarding the existence and uniqueness of solutions.

The dynamical system which we associate to (1.1) and investigate in this paper reads 
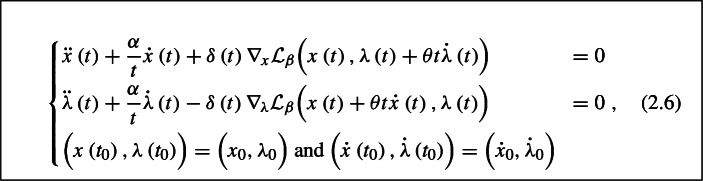
 where $t_0 >0$, $\alpha > 0$, $\theta > 0$, $\delta :\left[ t_{0}, +\infty \right) \rightarrow \mathbb {R}$ is a nonnegative continuously differentiable function and $\left( x_{0}, \lambda _{0} \right) , \bigl ( \dot{x}_{0}, \dot{\lambda }_{0} \bigr ) \in \mathcal {X}\times \mathcal {Y}$ are the initial conditions. Replacing the expressions of the partial gradients of $\mathcal {L}_{\beta }$ into the system leads to the following formulation for (2.6):The case (2.6) in which there is no time rescaling, i.e., when $\delta (t) \equiv 1$, was studied by Zeng et al. in [45], and by Boţ and Nguyen in [17]. The system with more general damping, extrapolation and time rescaling coefficients was addressed by He et al. in [27, 30] and by Attouch et al. in [10]. We mention that extending the results in this paper to the multi-block case is possible. For further details, we refer the readers to [17, Sect. 2.4].

It is well known that the viscous damping term $\frac{\alpha }{t}$ has a vital role in achieving fast convergence in unconstrained minimization [6, 8, 33]. The role of the extrapolation $\theta t$ is to induce more flexibility in the dynamical system and in the associated discrete schemes, as it has been recently noticed in [10, 12, 27, 45]. The time scaling function $\delta \left( \cdot \right)$ has the role to further improve the rates of convergence of the objective function value along the trajectory, as it was noticed in the context of unconstrained minimization problems in [7, 9, 11] and of linearly constrained minimization problems in [10, 30].

It is straightforward to show the existence of local solutions to (2.6), under the additional assumption that $\nabla f$ is Lipschitz continuous on every bounded subset of $\mathcal {X}$. First, notice that (2.6) can be rewritten as a first-order dynamical system. Indeed, $\left( x, \lambda \right) :\left[ t_{0}, + \infty \right) \rightarrow \mathcal {X} \times \mathcal {Y}$ is a solution to (2.6) if and only if $(x, \lambda , y, \nu ) :\left[ t_{0}, + \infty \right) \rightarrow \mathcal {X} \times \mathcal {Y} \times \mathcal {X} \times \mathcal {Y}$ is a solution towhere $F :\left[ t_{0}, + \infty \right) \times \mathcal {X} \times \mathcal {Y} \times \mathcal {X} \times \mathcal {Y} \rightarrow \mathcal {X} \times \mathcal {Y} \times \mathcal {X} \times \mathcal {Y}$ is given by

$$\begin{aligned}&F(t, z, \mu , w, \eta ) \\&\quad := \left( w, \eta , -\frac{\alpha }{t}w - \delta (t)\left[ \nabla f(z) + A^{*}\bigl ( \mu + \theta t \eta \bigr ) + \beta A^{*}\bigl ( Az - b\bigr )\right] ,\right. \\&\qquad \left. -\frac{\alpha }{t}\eta + \delta (t)\bigl [ A\bigl ( z + \theta t w\bigr ) - b\bigr ]\right) . \end{aligned}$$

where *F* is evidently continuous in *t*, and $F(t, \cdot )$ is Lipschitz continuous on every bounded subset, provided that the same property holds for $\nabla f$. We can then employ a theorem such as that by Cauchy-Lipschitz to obtain the existence of a unique solution to the previous system, and thus a unique solution to (2.6), defined on a maximal interval $[t_{0}, T_{\text {max}})$. To go further and show the existence and uniqueness of a global solution (that is, $T_{\text {max}} = +\infty$) we will need some energy estimates derived in the next section in a similar way as in [11, 17]. For this reason, the existence and uniqueness of a global solution is postponed to a later stage.

## Faster Convergence Rates via Time Rescaling

In this section we will derive fast convergence rates for the primal-dual gap, the feasibility measure, and the objective function value along the trajectories generated by the dynamical system (2.6). We will make the following assumptions on the parameters $\alpha$, $\theta$, $\beta$ and the function $\delta$ throughout this section. 
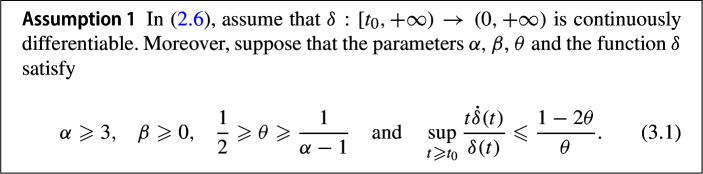
 Besides the first three conditions that are known previously in [17], it is worth pointing out that we can deduce from the last one the following inequality for every $t \geqslant t_{0}$:

3.2 $$\begin{aligned} \dfrac{t \dot{\delta } \left( t \right) }{\delta \left( t \right) } \leqslant \dfrac{1 - 2 \theta }{\theta } = \dfrac{1}{\theta } - 2 \leqslant \alpha - 3 . \end{aligned}$$

This gives a connection to the condition which appears in [7]. A few more comments regarding the function $\delta$ will come later, after the convergence rates statements.

### The Energy Function

Let $\left( x, \lambda \right) :\left[ t_{0}, + \infty \right) \rightarrow \mathcal {X}\times \mathcal {Y}$ be a solution of (2.6). Let $(x_{*}, \lambda _{*}) \in \mathbb {S}$ be fixed, we define the energy function $\mathcal {E}:\left[ t_{0}, + \infty \right) \rightarrow \mathbb {R}$

3.3 $$\begin{aligned} \mathcal {E}\left( t \right)&:= \theta ^{2} t^{2} \delta (t) \left( \mathcal {L}_{\beta }\left( x \left( t \right) , \lambda _{*} \right) - \mathcal {L}_{\beta }\left( x_{*}, \lambda \left( t \right) \right) \right) + \dfrac{1}{2} \left\Vert v \left( t \right) \right\Vert ^{2} \nonumber \\&\quad + \dfrac{\xi }{2} \left\Vert \bigl ( x \left( t \right) , \lambda \left( t \right) \bigr ) - (x_{*}, \lambda _{*}) \right\Vert ^{2}, \end{aligned}$$

where

3.4 $$\begin{aligned} v \left( t \right)&:= \bigl ( x \left( t \right) , \lambda \left( t \right) \bigr ) - (x_{*}, \lambda _{*}) + \theta t \left( \dot{x} \left( t \right) , \dot{\lambda } \left( t \right) \right) , \end{aligned}$$

3.5 $$\begin{aligned} \xi&:= \alpha \theta - \theta - 1 \geqslant 0 . \end{aligned}$$

Notice that, according to (2.4) and (2.5), we have for every $t \geqslant t_0$

3.6 $$\begin{aligned} \mathcal {L}_{\beta }\left( x \left( t \right) , \lambda _{*} \right) - \mathcal {L}_{\beta }\left( x_{*}, \lambda \left( t \right) \right)&= \mathcal {L}\left( x \left( t \right) , \lambda _{*} \right) - \mathcal {L}\left( x_{*}, \lambda \left( t \right) \right) + \dfrac{\beta }{2} \left\Vert Ax \left( t \right) - b \right\Vert ^{2} \end{aligned}$$

3.7 $$\begin{aligned}&= \mathcal {L}\left( x \left( t \right) , \lambda _{*} \right) - f_{*} + \dfrac{\beta }{2} \left\Vert Ax \left( t \right) - b \right\Vert ^{2} \nonumber \\&= f \left( x \left( t \right) \right) - f_{*} \nonumber \\&\quad + \left\langle \lambda _{*}, Ax \left( t \right) - b \right\rangle + \dfrac{\beta }{2} \left\Vert Ax \left( t \right) - b \right\Vert ^{2} \geqslant 0, \end{aligned}$$

where $f_{*}$ denotes the optimal objective value of (1.1). In addition, due to (3.7), we have

3.8 $$\begin{aligned} \mathcal {E}\left( t \right) \geqslant 0 \qquad \forall t \geqslant t_{0} . \end{aligned}$$

The construction of $\mathcal {E}$ is inspired by [17]. However, one can notice that we only consider $\mathcal {E}$ defined with respect to a fixed primal-dual solution $\left( x_{*}, \lambda _{*} \right) \in \mathbb {S}$ rather than a family of energy functions, each defined with respect to a point $\left( z, \mu \right) \in \mathbb {F} \times \mathcal {Y}$. This gives simpler proofs for some results when compared to those in [17].

Assumption 1 implies the nonnegativity of following quantity, which will appear many times in our analysis:

3.9 $$\begin{aligned} \sigma :\left[ t_{0}, + \infty \right) \rightarrow \mathbb {R}_{+}, \quad \sigma (t) := \frac{1 - 2\theta }{\theta }\delta (t) - t \dot{\delta }(t) . \end{aligned}$$

The following lemma gives us the decreasing property of the energy function. As a consequence of this lemma, we obtain some integrability results which will be needed later. The proofs are postponed to the Appendix.

#### Lemma 3.1

Let $\left( x, \lambda \right) :\left[ t_{0}, + \infty \right) \rightarrow \mathcal {X}\times \mathcal {Y}$ be a solution of (2.6) and $(x_{*}, \lambda _{*}) \in \mathbb {S}$. For every $t \geqslant t_{0}$ it holds

$$\begin{aligned} \dfrac{d}{dt} \mathcal {E}\left( t \right)&\leqslant - \theta ^{2} t \sigma (t) \left( \mathcal {L}_{\beta }\left( x \left( t \right) , \lambda _{*} \right) - \mathcal {L}_{\beta }\left( x_{*}, \lambda \left( t \right) \right) \right) - \dfrac{1}{2} \beta \theta t \delta \left( t \right) \left\Vert Ax \left( t \right) - b \right\Vert ^{2} \\&\quad - \xi \theta t \left\Vert \left( \dot{x} \left( t \right) , \dot{\lambda } \left( t \right) \right) \right\Vert ^{2} . \end{aligned}$$

#### Proof

See “Proof of Lemma 3.1” in Appendix B. $\square$

#### Theorem 3.2

Let $(x, \lambda ): \left[ t_{0}, +\infty \right) \rightarrow \mathcal {X} \times \mathcal {Y}$ be a solution of (2.6) and $(x_{*}, \lambda _{*}) \in \mathbb {S}$. The following statements are true

(i) it holds
  3.10 $$\begin{aligned} \int _{t_{0}}^{+\infty } t \sigma (t)\Big [\mathcal {L}\left( x(t), \lambda _{*} \right) - \mathcal {L}(x_{*}, \lambda (t))\Big ]dt&\leqslant \mathcal {E}(t_{0}) \,< +\infty , \end{aligned}$$
  3.11 $$\begin{aligned} \beta \int _{t_{0}}^{+\infty } t \delta (t) \left\Vert Ax \left( t \right) - b \right\Vert ^{2}dt&\leqslant \frac{2\mathcal {E}(t_{0})}{\theta } \,< +\infty , \end{aligned}$$
  3.12 $$\begin{aligned} \xi \int _{t_{0}}^{+\infty } t \left\Vert \left( \dot{x} \left( t \right) , \dot{\lambda } \left( t \right) \right) \right\Vert ^{2}&\leqslant \frac{\mathcal {E}(t_{0})}{\theta } \, < +\infty ; \end{aligned}$$
(ii) if, in addition, $\alpha > 3$ and $\frac{1}{2} \geqslant \theta > \frac{1}{\alpha - 1}$, then the trajectory $(x(t), \lambda (t))_{t\geqslant t_{0}}$ is bounded and the convergence rate of its velocity is
  $$\begin{aligned} \left\Vert \left( \dot{x} \left( t \right) , \dot{\lambda } \left( t \right) \right) \right\Vert = \mathcal {O}\left( \frac{1}{t}\right) \quad \text {as} \quad t\rightarrow +\infty . \end{aligned}$$

#### Proof

See “Proof of Theorem 3.2” in Appendix B. $\square$

### Fast Convergence Rates for the Primal-Dual Gap, the Feasibility Measure and the Objective Function Value

The following are the main convergence rates results of the paper.

#### Theorem 3.3

Let $(x, \lambda ): \left[ t_{0}, +\infty \right) \rightarrow \mathcal {X}\times \mathcal {Y}$ be a solution of (2.6) and $(x_{*}, \lambda _{*}) \in \mathbb {S}$. The following statements are true

(i) for every $t\geqslant t_{0}$ it holds
  3.13 $$\begin{aligned} 0 \leqslant \mathcal {L}\left( x(t), \lambda _{*} \right) - \mathcal {L}(x_{*}, \lambda (t)) \leqslant \frac{\mathcal {E}(t_{0})}{\theta ^{2} t^{2} \delta (t)}; \end{aligned}$$
(ii) for every $t\geqslant t_{0}$ it holds
  3.14 $$\begin{aligned} \left\Vert Ax \left( t \right) - b \right\Vert \leqslant \frac{2 C_{1}}{t^{2} \delta (t)}, \end{aligned}$$
  where
  $$\begin{aligned} C_{1}&:= \sup _{t\geqslant t_{0}} t \left\Vert \dot{\lambda } \left( t \right) \right\Vert + (\alpha - 1) \sup _{t\geqslant t_{0}} \left\Vert \lambda \left( t \right) - \lambda _{*} \right\Vert \\&\quad + t_{0}^{2} \delta (t_{0}) \left\Vert Ax(t_{0}) - b \right\Vert + t_{0}\left\Vert \dot{\lambda } \left( t_{0} \right) \right\Vert ; \end{aligned}$$
(iii) for every $t\geqslant t_{0}$ it holds
  3.15 $$\begin{aligned} \left|f \left( x \left( t \right) \right) - f_{*} \right|\leqslant \left( \frac{\mathcal {E}(t_{0})}{\theta ^{2}} + 2 C_{1} \left\Vert \lambda _{*} \right\Vert \right) \frac{1}{t^{2} \delta (t)}. \end{aligned}$$

#### Proof

(i) We have already established that $\mathcal {E}$ is nonincreasing on $\left[ t_{0}, +\infty \right)$. Therefore, from the expression for $\mathcal {E}$ and relation (3.6) we deduce

3.16 $$\begin{aligned} \theta ^{2} t^{2} \delta (t) \bigl [\mathcal {L}\left( x(t), \lambda _{*} \right) - \mathcal {L}(x_{*}, \lambda (t)) \bigr ] \leqslant \mathcal {E}(t_{0}) \quad \forall t\geqslant t_{0}, \end{aligned}$$

and the first claim follows.

(ii) From the second line of (2.6), for every $t\geqslant t_{0}$ we have

3.17 $$\begin{aligned} t \ddot{\lambda }(t) + \alpha \dot{\lambda }(t) = t\delta (t) \Bigl (A\bigl (x(t) + \theta t \dot{x}(t)\bigr ) - b\Bigr ) = t\delta (t)\bigl ( Ax(t) - b\bigr ) + \theta t^{2} \delta (t) A\dot{x}(t). \end{aligned}$$

Fix $t\geqslant t_{0}$. On the one hand, integration by parts yields

3.18 $$\begin{aligned} \begin{aligned} \int _{t_{0}}^{t}\bigl ( s \ddot{\lambda }(s) + \alpha \dot{\lambda }(s)\bigr )ds&= t \dot{\lambda }(t) - t_{0} \dot{\lambda }(t_{0}) - \int _{t_{0}}^{t}\dot{\lambda }(s)ds + \alpha \int _{t_{0}}^{t} \dot{\lambda }(s)ds \\&= t \dot{\lambda }(t) - t_{0} \dot{\lambda }(t_{0}) + (\alpha - 1)(\lambda (t) - \lambda (t_{0})). \end{aligned} \end{aligned}$$

On the other hand, again integrating by parts leads to

3.19 $$\begin{aligned} \begin{aligned} \int _{t_{0}}^{t}s^{2} \delta (s) A\dot{x}(s) ds&= t^{2} \delta (t) (Ax(t) - b) - t_{0}^{2} \delta (t_{0}) (Ax(t_{0}) - b) \\&- \int _{t_{0}}^{t}\bigl ( 2 s \delta (s) + s^{2} \dot{\delta }(s)\bigr ) (Ax(s) - b)ds. \end{aligned} \end{aligned}$$

Now, integrating (3.17) from $t_{0}$ to *t* and using (3.18) and (3.19) gives us

3.20 $$\begin{aligned}&t \dot{\lambda }(t) - t_{0} \dot{\lambda }(t_{0}) + (\alpha - 1)(\lambda (t) - \lambda (t_{0})) \nonumber \\&\quad = \int _{t_{0}}^{t}s \delta (s) (Ax(s) - b) ds + \theta \int _{t_{0}}^{t} s^{2} \delta (s) A\dot{x}(s) ds \nonumber \\&\quad = t^{2} \delta (t) (Ax(t) - b) - t_{0}^{2} \delta (t_{0})(Ax(t_{0}) - b) \nonumber \\&\qquad + \int _{t_{0}}^{t} s \bigl [(1 - 2\theta )\delta (s) - \theta s \dot{\delta }(s)\bigr ] (Ax(s) - b) ds \nonumber \\&\quad = t^{2} \delta (t) (Ax(t) - b) - t_{0}^{2} \delta (t_{0})(Ax(t_{0}) - b)\nonumber \\&\qquad + \int _{t_{0}}^{t}\frac{(1 - 2\theta )\delta (s) - \theta s \dot{\delta }(s)}{s \delta (s)} s^{2} \delta (s) (Ax(s) - b) ds. \end{aligned}$$

It follows that, for every $t \geqslant t_{0}$, we have

3.21 $$\begin{aligned} \left\| t^{2} \delta (t) (Ax(t) - b) + \int _{t_{0}}^{t}\frac{(1 - 2\theta )\delta (s) - \theta s \dot{\delta }(s)}{s \delta (s)} s^{2} \delta (s) (Ax(s) - b) ds \right\| \leqslant C_{1}, \end{aligned}$$

where

$$\begin{aligned} C_{1}= & {} \sup _{t\geqslant t_{0}} t\left\Vert \dot{\lambda } \left( t \right) \right\Vert + (\alpha - 1) \sup _{t\geqslant t_{0}}\Vert \lambda (t) - \lambda \left( t_{0} \right) \Vert + t_{0}^{2} \delta (t_{0}) \left\Vert Ax(t_{0}) - b \right\Vert \\{} & {} +\, t_{0}\left\Vert \dot{\lambda } \left( t_{0} \right) \right\Vert < + \infty , \end{aligned}$$

and this quantity is finite in light of (B.7) and (B.5). Now, we set

$$\begin{aligned} g(t):= t^{2} \delta (t) \left\Vert Ax \left( t \right) - b \right\Vert , \qquad a(t):= \frac{(1 - 2\theta )\delta (t) - \theta t \dot{\delta }(t)}{t \delta (t)} \qquad \forall t\geqslant t_{0} \end{aligned}$$

and we apply Lemma A.1 to deduce that

3.22 $$\begin{aligned} t^{2} \delta (t) \left\Vert Ax \left( t \right) - b \right\Vert \leqslant 2C_{1} \quad \forall t\geqslant t_{0}. \end{aligned}$$

(iii) For a fixed $t\geqslant t_{0}$, we have

$$\begin{aligned} \mathcal {L}\left( x(t), \lambda _{*} \right) - \mathcal {L}(x_{*}, \lambda (t)) = f(x(t)) - f(x_{*}) + \langle \lambda _{*}, Ax(t) - b\rangle . \end{aligned}$$

Therefore, from using (3.22) and (3.16) we obtain, for every $t\geqslant t_{0}$,

$$\begin{aligned} \left|f \left( x \left( t \right) \right) - f_{*} \right|&\leqslant \mathcal {L}\left( x(t), \lambda _{*} \right) - \mathcal {L}(x_{*}, \lambda (t)) + \left\Vert \lambda _{*} \right\Vert \left\Vert Ax \left( t \right) - b \right\Vert \\&\leqslant \left( \frac{\mathcal {E}(t_{0})}{\theta ^{2}} + 2C_{1} \left\Vert \lambda _{*} \right\Vert \right) \frac{1}{t^{2} \delta (t)}, \end{aligned}$$

which leads to the last statement. $\square$

Some comments regarding the previous proof and results are in order.

#### Remark 3.4

The proof we provided here is significantly shorter than the one derived in [17] thanks to Lemma A.1. This Lemma is the one used in [28] for showing the fast convergence to zero of the feasibility measure, although the authors study a different dynamical system. On the other hand, when $\delta \left( t \right) \equiv 1$, the result in [17] is more robust than the one we obtain here, as it gives the $\mathcal {O}\left( \frac{1}{t^{2}} \right)$ rates for the sum of primal-dual gap and feasibility measure, rather than each one individually. It also allows us to focus only on the energy function defined with respect to a primal-dual optimal solution $\left( x_{*}, \lambda _{*} \right) \in \mathbb {S}$, rather than on an arbitrary feasible point $\left( z, \mu \right) \in \mathbb {F} \times \mathcal {Y}$ as in [17].

#### Remark 3.5

Here are some remarks comparing our rates of convergence to those in [10, 30].

- *Primal-dual gap*: According to (3.13), the following rate of convergence for the primal-dual is exhibited:
  $$\begin{aligned} \mathcal {L}\bigl (x(t), \lambda _{*}\bigr ) - \mathcal {L}\bigl (x_{*}, \lambda (t)\bigr ) = \mathcal {O}\left( \frac{1}{t^{2} \delta (t)}\right) \quad \text {as} \quad t\rightarrow +\infty , \end{aligned}$$
  which coincides with the findings of [10, 30].
- *Feasibility measure*: According to (3.14), we have
  $$\begin{aligned} \left\Vert Ax \left( t \right) - b \right\Vert = \mathcal {O}\left( \frac{1}{t^{2} \delta (t)}\right) \quad \text {as} \quad t\rightarrow +\infty , \end{aligned}$$
  which improves the rate $\mathcal {O}\left( \frac{1}{t \sqrt{\delta (t)}}\right)$ reported in [10, 30].
- *Functional values*: Relation (3.15) tells us that
  $$\begin{aligned} \left|f \left( x \left( t \right) \right) - f_{*} \right|= \mathcal {O}\left( \frac{1}{t^{2} \delta (t)}\right) \quad \text {as} \quad t\rightarrow +\infty . \end{aligned}$$
  In [10], only the upper bound presents this order of convergence. The lower bound obtained is of order $\mathcal {O}\left( \frac{1}{t \sqrt{\delta (t)}}\right)$ as $t \rightarrow +\infty$. In [30], there are no comments on the rate attained by the functional values in the case of a general time rescaling parameter.
- *Further comparisons with*  [30]: in [30, Theorem 2.16], unlike the preceding result [30, Theorem 2.15], the authors produce a rate of $\mathcal {O}\left( \frac{1}{t^{1/\theta }}\right)$ as $t\rightarrow +\infty$ for $\Vert Ax(t) - b\Vert$ and $|f(x(t)) - f(x_{*})|$, provided the time rescaling parameter is chosen to be $\delta (t) = \delta _{0} t^{\frac{1}{\theta } - 2}$, for some $\delta _{0} > 0$. This choice comes from the solution to the differential equation
  $$\begin{aligned} \frac{t \dot{\delta }(t)}{\delta (t)} = \frac{1 - 2\theta }{\theta } \quad \forall t\geqslant t_{0}, \end{aligned}$$
  and thus is covered by our results when the growth condition (3.1) holds with equality. The rates are consequently
  $$\begin{aligned} \mathcal {O}\left( \frac{1}{t^{2} \cdot \delta _{0} t^{\frac{1}{\theta } - 2}}\right) = \mathcal {O}\left( \frac{1}{t^{\frac{1}{\theta }}}\right) \quad \text {as} \quad t\rightarrow +\infty . \end{aligned}$$
  In this setting, if we wish to obtain fast convergence rates, we need to choose a small $\theta$. In light of Assumption 1, where we have $\frac{1}{2}\geqslant \theta \geqslant \frac{1}{\alpha - 1}$, this can be achieved by taking $\alpha$ large enough. Such behaviour can also be seen in [10] and in the unconstrained case [7, 11].

## Weak Convergence of the Trajectory to a Primal-Dual Solution

In this section we will show that the solutions to (2.6) weakly converge to an element of $\mathbb {S}$. The fact that $\delta \left( t \right)$ enters the convergence rate statement suggests that one can benefit from this time rescaling function when it is at least nondecreasing on $\left[ t_{0}, +\infty \right)$. We are, in fact, going to need this condition when showing trajectory convergence. 
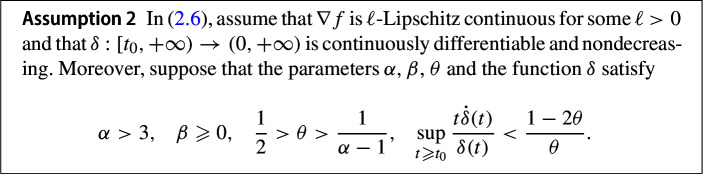


Assumption 2 entails the existence of $C_{2} > 0$ such that

4.1 $$\begin{aligned} \frac{t \dot{\delta }(t)}{\delta (t)} + C_{2} \leqslant \frac{1 - 2\theta }{\theta } \quad \forall t \geqslant t_{0}. \end{aligned}$$

and therefore it follows further from the nondecreasing property of $\delta$ that

4.2 $$\begin{aligned} 0 < C_{2} \delta (t_{0}) \leqslant C_{2} \delta (t) \leqslant (1 - 2\theta )\delta (t) - \theta t \dot{\delta }(t) \quad \forall t\geqslant t_{0} . \end{aligned}$$

Moreover, from (4.1), for every $t\geqslant t_{0}$, we have

$$\begin{aligned} 0 < C_{2} \leqslant \frac{1 - 2\theta }{\theta } - \frac{t \dot{\delta }(t)}{\delta (t)} = \frac{\sigma (t)}{\delta (t)}, \end{aligned}$$

which gives

4.3 $$\begin{aligned} \delta (t) \leqslant \frac{\sigma (t)}{C_{2}} \quad \forall t\geqslant t_{0}. \end{aligned}$$

We can now formally state the existence and uniqueness result of the trajectory. The proof follows the same argument as in [17, Theorem 4.1], therefore we omit the details.

### Theorem 4.1

In the setting of Assumption 2, for every choice of initial conditions

$$\begin{aligned} x(t_{0}) = x_{0}, \quad \lambda (t_{0}) = \lambda _{0}, \quad \dot{x}(t_{0}) = \dot{x}_{0}, \quad \text {and} \quad \dot{\lambda }(t_{0}) = \dot{\lambda }_{0} \end{aligned}$$

the system (2.6) has a unique global twice continuously differentiable solution $(x, \lambda ): \left[ t_{0}, +\infty \right) \rightarrow \mathcal {X} \times \mathcal {Y}$.

The additional Lipschitz continuity condition of $\nabla f$ and the fact that $\delta$ is nondecreasing give rise to the following two essential integrability statements.

### Proposition 4.2

Let $(x, \lambda ): \left[ t_{0}, +\infty \right) \rightarrow \mathcal {X} \times \mathcal {Y}$ be a solution of (2.6) and $(x_{*}, \lambda _{*}) \in \mathbb {S}$. Then it holds

4.4 $$\begin{aligned} \int _{t_{0}}^{+\infty }t\delta (t) \left\| \nabla f(x(t)) - \nabla f(x_{*})\right\| ^{2} dt < +\infty \end{aligned}$$

and

4.5 $$\begin{aligned} \int _{t_{0}}^{+\infty } t\delta (t) \left\Vert Ax \left( t \right) - b \right\Vert ^{2} dt < +\infty . \end{aligned}$$

### Proof

See “Proof of Proposition 4.2” in Appendix B. $\square$

Now, for a given primal-dual solution $(x_{*}, \lambda _{*}) \in \mathbb {S}$, we define the following mappings on $\left[ t_{0}, +\infty \right)$

4.6 $$\begin{aligned} W(t)&:= \delta (t) \bigl [\mathcal {L}_{\beta }\left( x(t), \lambda _{*} \right) - \mathcal {L}_{\beta }(x_{*}, \lambda (t))\bigr ] + \frac{1}{2}\left\Vert \left( \dot{x} \left( t \right) , \dot{\lambda } \left( t \right) \right) \right\Vert ^{2} \geqslant 0, \end{aligned}$$

4.7 $$\begin{aligned} \varphi (t)&:= \frac{1}{2}\left\| \bigl (x(t), \lambda (t)\bigr ) - (x_{*}, \lambda _{*})\right\| ^{2} \geqslant 0. \end{aligned}$$

The following are three technical lemmas that we will need in this section. Lemma 4.4 guarantess that the first condition of Opial’s Lemma is met.

### Lemma 4.3

Let $(x, \lambda ): \left[ t_{0}, +\infty \right) \rightarrow \mathcal {X} \times \mathcal {Y}$ a solution of (2.6) and $(x_{*}, \lambda _{*}) \in \mathbb {S}$. The following inequality holds for every $t\geqslant t_{0}$:

4.8 $$\begin{aligned} \ddot{\varphi }(t) + \frac{\alpha }{t} \dot{\varphi }(t) + \theta t \dot{W}(t) + \frac{\delta (t)}{2\ell }\left\Vert \nabla f \left( x \left( t \right) \right) - \nabla f \left( x_{*} \right) \right\Vert ^{2} + \frac{\beta \delta (t)}{2}\left\Vert Ax \left( t \right) - b \right\Vert ^{2} \leqslant 0. \end{aligned}$$

### Proof

See “Proof of Lemma 4.3” in Appendix B. $\square$

### Lemma 4.4

Let $(x, \lambda ): \left[ t_{0}, +\infty \right) \rightarrow \mathcal {X} \times \mathcal {Y}$ be a solution to (2.6) and $(x_{*}, \lambda _{*}) \in \mathbb {S}$. Then the positive part $[\dot{\varphi }]_{+}$ of $\dot{\varphi }$ belongs to $\mathbb {L}^{1}\left[ t_{0}, +\infty \right)$ and the limit $\lim _{t \rightarrow +\infty } \varphi (t)$ exists.

### Proof

For any $t\geqslant t_{0}$, we multiply (4.8) by *t* and drop the last two norm squared terms to obtain

$$\begin{aligned} t \ddot{\varphi }(t) + \alpha \dot{\varphi }(t) + \theta t^{2} \dot{W}(t) \leqslant 0. \end{aligned}$$

Recall from (4.6) that for every $t\geqslant t_{0}$ we have

4.9 $$\begin{aligned} tW(t) = t\delta (t) \bigl [\mathcal {L}_{\beta }\left( x(t), \lambda _{*} \right) - \mathcal {L}_{\beta }(x_{*}, \lambda (t))\bigr ] + \frac{t}{2}\left\Vert \left( \dot{x} \left( t \right) , \dot{\lambda } \left( t \right) \right) \right\Vert ^{2}. \end{aligned}$$

On the one hand, according to (3.12), the second summand of the previous expression belongs to $\mathbb {L}^{1}\left[ t_{0}, +\infty \right)$. On the other hand, using (4.3) and (3.10), we assert that

$$\begin{aligned}{} & {} \int _{t_{0}}^{+\infty } t \delta (t) \bigl [\mathcal {L}_{\beta }\left( x(t), \lambda _{*} \right) - \mathcal {L}_{\beta }(x_{*}, \lambda (t))\bigr ] dt\\{} & {} \quad \leqslant \frac{1}{C_{2}} \int _{t_{0}}^{+\infty } t \sigma (t) \bigl [\mathcal {L}_{\beta }\left( x(t), \lambda _{*} \right) - \mathcal {L}_{\beta }(x_{*}, \lambda (t))\bigr ] dt < +\infty . \end{aligned}$$

Hence, the first summand of (4.9) also belongs to $\mathbb {L}^{1}\left[ t_{0}, +\infty \right)$, which implies that the mapping $t \mapsto tW(t)$ belongs to $\mathbb {L}^{1}\left[ t_{0}, +\infty \right)$ as well. For achieving the desired conclusion, we make use of Lemma A.4 with $\phi := \varphi$ and $w:= \theta W$. $\square$

### Lemma 4.5

Let $(x, \lambda ): \left[ t_{0}, +\infty \right) \rightarrow \mathcal {X} \times \mathcal {Y}$ be a solution to (2.6) and $(x_{*}, \lambda _{*}) \in \mathbb {S}$. The following inequality holds for every $t\geqslant t_{0}$

$$\begin{aligned}&\frac{\alpha }{t \delta (t)} \frac{d}{dt}\left\Vert \left( \dot{x} \left( t \right) , \dot{\lambda } \left( t \right) \right) \right\Vert ^{2} + 2\left\langle \ddot{x}(t) + \frac{\alpha }{t}\dot{x}(t), A^{*}(\lambda (t) - \lambda _{*})\right\rangle \\&\quad +\theta \frac{d}{dt}\Bigl (t \delta (t) \left\| A^{*}(\lambda (t) - \lambda _{*})\right\| ^{2}\Bigr ) + \bigl ((1 - \theta )\delta (t) - \theta t \dot{\delta }(t)\bigr ) \left\| A^{*}(\lambda (t) - \lambda _{*})\right\| ^{2} \\&\quad \leqslant \delta (t) \Bigl [ 2\Vert \nabla f(x(t)) - \nabla f(x_{*})\Vert ^{2} + \left( 2\beta ^{2} \Vert A\Vert ^{2} + 1\right) \left\Vert Ax \left( t \right) - b \right\Vert ^{2}\Bigr ]. \end{aligned}$$

### Proof

See “Proof of Lemma 4.5” in Appendix B. $\square$

### Lemma 4.6

Let $(x, \lambda ): [t_{0}, +\infty ) \rightarrow \mathcal {X} \times \mathcal {Y}$ be a solution to (2.6) and $(x_{*}, \lambda _{*}) \in \mathbb {S}$. Then, for every $t\geqslant t_{0}$ it holds

4.10 $$\begin{aligned}&\theta t^{\alpha + 1} \delta (t) \left\Vert A^{*} \left( \lambda \left( t \right) - \lambda _{*} \right) \right\Vert ^{2} \nonumber \\&\quad \leqslant -t^{\alpha } \dot{\varphi }(t) + \int _{t_{0}}^{t} s^{\alpha } V(s) ds \nonumber \\&\qquad + \int _{t_{0}}^{t} s^{\alpha } \Bigl [ \bigl (\theta (\alpha + 1) - 1\bigr )\delta (t) + \theta s \dot{\delta }(s)\Bigr ] \left\Vert A^{*}(\lambda (s) - \lambda _{*}) \right\Vert ^{2} ds \nonumber \\&\qquad -2 t^{\alpha } \bigl \langle \dot{x}(t), A^{*}(\lambda (t) - \lambda _{*})\bigr \rangle + C_{5}, \end{aligned}$$

where, for $$s\geqslant t_{0}$$,

$$\begin{aligned} V(s)&:= \theta (\alpha + 1) W(s) + \left( \frac{\alpha (\alpha - 1)}{t_{0}^{2} \delta (_{0})} + \left\Vert A \right\Vert \right) \bigl \Vert \bigl ( \dot{x}(s), \dot{\lambda }(s)\bigr )\bigr \Vert ^{2} \\&\quad + C_{3} \delta (s) \Vert \nabla f(x(s)) - \nabla f(x_{*})\Vert ^{2} + C_{4} \delta (s) \Vert Ax(s) - b\Vert ^{2}, \end{aligned}$$

for certain nonnegative constants $C_{3}, C_{4}$ and $C_{5}$.

### Proof

See “Proof of Lemma 4.6” in Appendix B. $\square$

The following proposition provides us with the main integrability result that will be used for verifying the second condition of Opial’s Lemma.

### Proposition 4.7

Let $(x, \lambda ): \left[ t_{0}, +\infty \right) \rightarrow \mathcal {X} \times \mathcal {Y}$ be a solution to (2.6) and $(x_{*}, \lambda _{*}) \in \mathbb {S}$. Then it holds

4.11 $$\begin{aligned} \int _{t_{0}}^{+\infty } t \delta (t) \left\Vert A^{*} \left( \lambda \left( t \right) - \lambda _{*} \right) \right\Vert ^{2} dt < +\infty . \end{aligned}$$

### Proof

We divide (4.10) by $t^{\alpha }$, thus obtaining

$$\begin{aligned} \theta t \delta (t) \left\Vert A^{*} \left( \lambda \left( t \right) - \lambda _{*} \right) \right\Vert ^{2}&\leqslant - \dot{\varphi }(t) + \frac{1}{t^{\alpha }}\int _{t_{0}}^{t} s^{\alpha } V(s) ds \\&\quad + \frac{1}{t^{\alpha }}\int _{t_{0}}^{t} s^{\alpha } \Bigl [ \bigl (\theta (\alpha + 1) - 1\bigr )\delta (s) + \theta s \dot{\delta }(s)\Bigr ] \\&\quad \left\Vert A^{*}(\lambda (s) - \lambda _{*}) \right\Vert ^{2} ds \\&\quad -2 \bigl \langle \dot{x}(t), A^{*}(\lambda (t) - \lambda _{*})\bigr \rangle + \frac{C_{5}}{t^{\alpha }}. \end{aligned}$$

Now, we integrate this inequality from $t_{0}$ to *r*. We get

4.12 $$\begin{aligned}&\theta \int _{t_{0}}^{r} t \delta (t) \left\Vert A^{*} \left( \lambda \left( t \right) - \lambda _{*} \right) \right\Vert ^{2} dt \nonumber \\&\quad \leqslant \ \varphi (t_{0}) - \varphi (r) + \int _{t_{0}}^{r}\frac{1}{t^{\alpha }} \left( \int _{t_{0}}^{t} s^{\alpha } V(s) ds\right) dt \nonumber \\&\qquad + \int _{t_{0}}^{r} \frac{1}{t^{\alpha }} \left( \int _{t_{0}}^{t} s^{\alpha } \Bigl [ \bigl (\theta (\alpha + 1) - 1\bigr )\delta (s) + \theta s \dot{\delta }(s)\Bigr ] \left\Vert A^{*}(\lambda (s) - \lambda _{*}) \right\Vert ^{2} ds\right) dt \nonumber \\&\qquad - 2 \int _{t_{0}}^{r} \bigl \langle A\dot{x}(t), \lambda (t) - \lambda _{*}\bigr \rangle dt + C_{5} \int _{t_{0}}^{r} t^{\alpha } dt. \end{aligned}$$

We now recall some important facts. First of all, we have

4.13 $$\begin{aligned} \int _{t_{0}}^{r}\frac{1}{t^{\alpha }} dt \leqslant \frac{1}{(\alpha - 1)t_{0}^{\alpha - 1}}. \end{aligned}$$

In addition, according to Lemma A.2, it holds

4.14 $$\begin{aligned} \int _{t_{0}}^{r} \frac{1}{t^{\alpha }} \left( \int _{t_{0}}^{t} s^{\alpha } V(s) ds\right) dt \leqslant \frac{1}{\alpha - 1}\int _{t_{0}}^{r}t V(t) dt, \end{aligned}$$

and

4.15 $$\begin{aligned}&\int _{t_{0}}^{r} \frac{1}{t^{\alpha }} \left( \int _{t_{0}}^{t} s^{\alpha } \Bigl [ \bigl (\theta (\alpha + 1) - 1\bigr )\delta (s) + \theta s \dot{\delta }(s)\Bigr ] \left\Vert A^{*}(\lambda (s) - \lambda _{*}) \right\Vert ^{2} ds\right) dt \nonumber \\&\quad \leqslant \frac{1}{\alpha - 1}\int _{t_{0}}^{r} \Bigl [ \bigl (\theta (\alpha + 1) - 1\bigr )\delta (t) + \theta t \dot{\delta }(t)\Bigr ] \left\Vert A^{*}(\lambda (t) - \lambda _{*}) \right\Vert ^{2} dt, \end{aligned}$$

respectively.

Finally, integrating by parts leads to

4.16 $$\begin{aligned}&- \int _{t_{0}}^{r} \bigl \langle A \dot{x}(t), \lambda (t) - \lambda _{*}\bigr \rangle dt \nonumber \\&\quad = \ - \bigl \langle Ax(r) - b, \lambda (r) - \lambda _{*}\bigr \rangle + \bigl \langle Ax(t_{0}) - b, \lambda (t_{0}) - \lambda _{*}\bigr \rangle + \int _{t_{0}}^{r} \bigl \langle Ax(t) - b, \dot{\lambda }(t)\bigr \rangle dt \nonumber \\&\quad \leqslant \Vert Ax(r) - b\Vert \Vert \lambda (r) - \lambda _{*}\Vert + \left\Vert Ax(t_{0}) - b \right\Vert \Vert \lambda (t_{0}) - \lambda _{*}\Vert + \int _{t_{0}}^{r} \bigl \langle Ax(t) - b, \dot{\lambda }(t)\bigr \rangle dt \nonumber \\&\quad \leqslant \sup _{t\geqslant t_{0}} \{\left\Vert Ax \left( t \right) - b \right\Vert \left\Vert \lambda \left( t \right) - \lambda _{*} \right\Vert \} + \left\Vert Ax(t_{0}) - b \right\Vert \Vert \lambda (t_{0} - \lambda _{*})\Vert \nonumber \\&\qquad + \frac{1}{2}\int _{t_{0}}^{r} \bigl ( \left\Vert Ax \left( t \right) - b \right\Vert ^{2} + \left\Vert \dot{\lambda } \left( t \right) \right\Vert ^{2}\bigr ) dt. \end{aligned}$$

The supremum term is finite due to the boundedness of the trajectory. Now, by using the nonnegativity of $\varphi$ and the facts (4.13), (4.14), (4.15) and (4.16) on (4.12), we come to

4.17 $$\begin{aligned}&\dfrac{\theta }{\alpha - 1} \int _{t_{0}}^{r}t \sigma (t) \left\Vert A^{*} \left( \lambda \left( t \right) - \lambda _{*} \right) \right\Vert ^{2} dt \nonumber \\&\quad = \int _{t_{0}}^{r} \left[ \theta \delta (t) - \frac{\bigl (\theta (\alpha + 1) - 1\bigr )\delta (t) + \theta t \dot{\delta }(t)}{\alpha - 1}\right] t \left\Vert A^{*} \left( \lambda \left( t \right) - \lambda _{*} \right) \right\Vert ^{2} dt \nonumber \\&\quad \leqslant \frac{1}{\alpha - 1}\int _{t_{0}}^{r} t V(t) dt + \int _{t_{0}}^{r} t \left( \left\Vert Ax \left( t \right) - b \right\Vert ^{2} + \left\Vert \dot{\lambda } \left( t \right) \right\Vert ^{2} \right) dt + C_{6}, \end{aligned}$$

where

$$\begin{aligned} C_{6}&:= \varphi (t_{0}) + 2 \sup _{t\geqslant t_{0}} \left\{ \left\Vert Ax \left( t \right) - b \right\Vert \left\Vert \lambda \left( t \right) - \lambda _{*} \right\Vert \right\} \\&\quad + 2 \left\Vert Ax(t_{0}) - b \right\Vert \Vert \lambda (t_{0} - \lambda _{*})\Vert + \frac{C_{5}}{(\alpha - 1)t_{0}^{\alpha - 1}}. \end{aligned}$$

According to (3.11) and (3.12) in Theorem 3.2, as well as Lemma 4.4, we know that the mappings $t \mapsto t V(t)$ and $t \mapsto t \left( \left\Vert Ax \left( t \right) - b \right\Vert ^{2} + \left\Vert \dot{\lambda } \left( t \right) \right\Vert \right)$ belong to $\mathbb {L}^{1}\left[ t_{0}, +\infty \right)$. Therefore, by taking the limit as $r \rightarrow +\infty$ in (4.17) we obtain

$$\begin{aligned} \int _{t_{0}}^{+\infty } t \sigma (t) \left\Vert A^{*} \left( \lambda \left( t \right) - \lambda _{*} \right) \right\Vert ^{2} dt < +\infty . \end{aligned}$$

Again, from (4.3) we conclude that

$$\begin{aligned} \int _{t_{0}}^{+\infty } t \delta (t) \left\Vert A^{*} \left( \lambda \left( t \right) - \lambda _{*} \right) \right\Vert ^{2} dt \leqslant \frac{1}{C_{2}} \int _{t_{0}}^{+\infty } t \sigma (t) \left\Vert A^{*} \left( \lambda \left( t \right) - \lambda _{*} \right) \right\Vert ^{2} dt < +\infty , \end{aligned}$$

which completes the proof. $\square$

The following result is the final step towards the second condition of Opial’s Lemma.

### Theorem 4.8

Let $(x, \lambda ): \left[ t_{0}, +\infty \right) \rightarrow \mathcal {X} \times \mathcal {Y}$ be a solution to (2.6) and $(x_{*}, \lambda _{*}) \in \mathbb {S}$. Then it holds

4.18 $$\begin{aligned}{} & {} \left\Vert \nabla f \left( x \left( t \right) \right) - \nabla f \left( x_{*} \right) \right\Vert = o \left( \frac{1}{\sqrt{t} \root 4 \of {\delta (t)}}\right) \text { and } \nonumber \\{} & {} \left\Vert A^{*} \left( \lambda \left( t \right) - \lambda _{*} \right) \right\Vert = o \left( \frac{1}{\sqrt{t} \root 4 \of {\delta (t)}}\right) \text { as } t \rightarrow +\infty . \end{aligned}$$

Consequently,

$$\begin{aligned} \left\Vert \nabla _{x} \mathcal {L}\bigl ( x \left( t \right) , \lambda \left( t \right) \bigr ) \right\Vert = \left\Vert \nabla f \left( x \left( t \right) \right) + A^{*} \lambda \left( t \right) \right\Vert = o\left( \frac{1}{\sqrt{t} \root 4 \of {\delta (t)}}\right) \quad \text {as} \quad t\rightarrow +\infty , \end{aligned}$$

while, as seen earlier,

$$\begin{aligned} \left\Vert \nabla _{\lambda } \mathcal {L}\bigl ( x \left( t \right) , \lambda \left( t \right) \bigr ) \right\Vert = \left\Vert Ax \left( t \right) - b \right\Vert = \mathcal {O}\left( \frac{1}{t^{2} \delta (t)}\right) \quad \text {as} \quad t\rightarrow +\infty . \end{aligned}$$

### Proof

We first show the gradient rate. For $t\geqslant t_{0}$, it holds

4.19 $$\begin{aligned}&\frac{d}{dt}\Bigl (t \sqrt{\delta (t)} \left\Vert \nabla f \left( x \left( t \right) \right) - \nabla f \left( x_{*} \right) \right\Vert ^{2}\Bigr )\nonumber \\&\quad = \left( \sqrt{\delta (t)} + \frac{t\dot{\delta }(t)}{2\sqrt{\delta (t)}}\right) \left\Vert \nabla f \left( x \left( t \right) \right) - \nabla f \left( x_{*} \right) \right\Vert ^{2} \nonumber \\&\quad + 2 t \sqrt{\delta (t)} \left\langle \nabla f(x(t)) - \nabla f(x_{*}), \frac{d}{dt}\nabla f(x(t))\right\rangle . \end{aligned}$$

On the one hand, by Assumption 2, we can write

4.20 $$\begin{aligned} \left( \sqrt{\delta (t)} + \frac{t\dot{\delta }(t)}{2\sqrt{\delta (t)}}\right)= & {} \left( \sqrt{\delta (t)} + \frac{\sqrt{\delta (t)}}{2} \cdot \frac{t\dot{\delta }(t)}{\delta (t)}\right) \leqslant \left( 1 + \frac{1 - 2\theta }{2\theta }\right) \sqrt{\delta (t)} \nonumber \\ {}= & {} \frac{1}{2\theta } \sqrt{\delta (t)}. \end{aligned}$$

Since $\delta$ is nondecreasing, for $t \geqslant t_{0}$ we have $\sqrt{\delta (t)} \geqslant \sqrt{\delta (t_{0})} > 0$. Set $t_{1}:= \max \left\{ t_{0}, \frac{1}{\sqrt{t_{0}}}\right\}$. Therefore, for $t \geqslant t_{1}$ it holds

$$\begin{aligned} \frac{1}{\sqrt{\delta (t)}} \leqslant \frac{1}{\sqrt{\delta (t_{0})}} = t_{1} \leqslant t \end{aligned}$$

and thus

4.21 $$\begin{aligned} \sqrt{\delta (t)} \leqslant t \delta (t). \end{aligned}$$

On the other hand, for every $t \geqslant t_{1}$ we deduce

4.22 $$\begin{aligned}&2 t \sqrt{\delta (t)} \left\langle \nabla f(x(t)) - \nabla f(x_{*}), \frac{d}{dt}\nabla f(x(t))\right\rangle \nonumber \\&\quad = 2 t \left\langle \sqrt{\delta (t)} \bigl [\nabla f(x(t)) - \nabla f(x_{*})\bigr ], \frac{d}{dt}\nabla f(x(t))\right\rangle \nonumber \\&\quad \leqslant t\delta (t) \left\Vert \nabla f \left( x \left( t \right) \right) - \nabla f \left( x_{*} \right) \right\Vert ^{2} + t\left\| \frac{d}{dt}\nabla f(x(t))\right\| ^{2} \nonumber \\&\quad \leqslant t\delta (t) \left\Vert \nabla f \left( x \left( t \right) \right) - \nabla f \left( x_{*} \right) \right\Vert ^{2} + \ell ^{2} t \bigl \Vert \dot{x}(t)\bigr \Vert ^{2} , \end{aligned}$$

where the last inequality is a consequence of the $\ell$-Lipschitz continuity of $\nabla f$. By combining (4.20), (4.21) and (4.22), from (4.19) we assert that for every $t\geqslant t_{1}$

$$\begin{aligned}{} & {} \frac{d}{dt}\Bigl (t \sqrt{\delta (t)} \left\Vert \nabla f \left( x \left( t \right) \right) - \nabla f \left( x_{*} \right) \right\Vert ^{2}\Bigr ) \\{} & {} \quad \leqslant \left( 1 + \frac{1}{2\theta } \right) t \delta (t) \left\Vert \nabla f \left( x \left( t \right) \right) - \nabla f \left( x_{*} \right) \right\Vert ^{2} + \ell ^{2} t \bigl \Vert \dot{x}(t)\bigr \Vert ^{2}. \end{aligned}$$

The right hand side of the previous inequality belongs to $\mathbb {L}^{1} [t_{1}, +\infty )$, according to (3.12) and (4.4). Since $\delta$ is nondecreasing, for every $t \geqslant t_{1}$ we have

$$\begin{aligned} \sqrt{\delta (t)} = \sqrt{\delta (t)} \cdot \frac{\sqrt{\delta (t)}}{\sqrt{\delta (t)}} \leqslant \frac{\delta (t)}{\sqrt{\delta (t_{0})}}, \end{aligned}$$

and thus

4.23 $$\begin{aligned}{} & {} \int _{t_{1}}^{+\infty } t \sqrt{\delta (t)} \left\Vert \nabla f \left( x \left( t \right) \right) - \nabla f \left( x_{*} \right) \right\Vert ^{2} dt \nonumber \\{} & {} \quad \leqslant \frac{1}{\sqrt{\delta (t_{0})}} \int _{t_{1}}^{+\infty } t \delta (t) \left\Vert \nabla f \left( x \left( t \right) \right) - \nabla f \left( x_{*} \right) \right\Vert ^{2} dt < +\infty . \end{aligned}$$

This means that the function being differentiated also belongs to $\mathbb {L}^{1} [t_{1}, +\infty )$. Therefore, Lemma A.3 gives us

$$\begin{aligned} t \sqrt{\delta (t)} \left\Vert \nabla f \left( x \left( t \right) \right) - \nabla f \left( x_{*} \right) \right\Vert ^{2} \rightarrow 0 \quad \text {as} \quad t\rightarrow +\infty . \end{aligned}$$

Proceeding in the exact same way, for every $t\geqslant t_{1}$ we have

$$\begin{aligned}&\frac{d}{dt} \Bigl ( t \sqrt{\delta (t)} \bigl \Vert A^{*}(\lambda (t) - \lambda _{*})\bigr \Vert ^{2}\Bigr ) \\&\quad = \left( \sqrt{\delta (t)} + \frac{t \dot{\delta }(t)}{2\sqrt{\delta (t)}}\right) \bigl \Vert A^{*}(\lambda (t) - \lambda _{*})\bigr \Vert ^{2} + 2 t \sqrt{\delta (t)} \bigl \langle A A^{*}(\lambda (t) - \lambda _{*}), \dot{\lambda }(t)\bigr \rangle \\&\quad \leqslant \left( \frac{1}{2\theta } + \Vert A\Vert ^{2} \right) t \delta (t) \bigl \Vert A^{*}(\lambda (t) - \lambda _{*})\bigr \Vert ^{2} + t \left\Vert \dot{\lambda } \left( t \right) \right\Vert ^{2}. \end{aligned}$$

According to (3.12) and (4.11), the right hand side of the previous inequality belongs to $\mathbb {L}^{1}[t_{1}, +\infty )$. Arguing as in (4.23), we deduce that the function being differentiated also belongs to $\mathbb {L}^{1}[t_{1}, +\infty )$. Again applying Lemma A.3, we come to

$$\begin{aligned} t \sqrt{\delta (t)} \bigl \Vert A^{*}(\lambda (t) - \lambda _{*})\bigr \Vert ^{2} \rightarrow 0 \quad \text {as} \quad t\rightarrow +\infty . \end{aligned}$$

Finally, recalling that $A^{*}\lambda _{*} = -\nabla f(x_{*})$, we deduce from the triangle inequality that

$$\begin{aligned} \left\Vert \nabla _{x} \mathcal {L}\bigl ( x \left( t \right) , \lambda \left( t \right) \bigr ) \right\Vert&= \left\Vert \nabla f \left( x \left( t \right) \right) + A^{*} \lambda \left( t \right) \right\Vert \\&\leqslant \left\Vert \nabla f \left( x \left( t \right) \right) - \nabla f \left( x_{*} \right) \right\Vert + \bigl \Vert A^{*}(\lambda (t) - \lambda _{*})\bigr \Vert \nonumber \\&= o\left( \frac{1}{\sqrt{t} \root 4 \of {\delta (t)}}\right) \quad \text {as} \quad t\rightarrow +\infty , \end{aligned}$$

and the third claim follows. $\square$

### Remark 4.9

The previous theorem also has its own interest. It tells us that the time rescaling parameter also plays a role in accelerating the rates of convergence for $\left\Vert \nabla f \left( x \left( t \right) \right) - \nabla f \left( x_{*} \right) \right\Vert$ and $\Vert A^{*}(\lambda (t) - \lambda _{*})\Vert$ as $t\rightarrow +\infty$. Moreover, we deduce from (4.18) that the mapping $(x, \lambda ) \mapsto (\nabla f(x), A^{*}\lambda )$ is constant along $\mathbb {S}$, as reported in [17, Proposition A.4].

We now come to the final step and show weak convergence of the trajectories of (2.6) to elements of $\mathbb {S}$.

### Theorem 4.10

Let $(x, \lambda ): \left[ t_{0}, +\infty \right) \rightarrow \mathcal {X} \times \mathcal {Y}$ be a solution to (2.6) and $\left( x_{*}, \lambda _{*} \right) \in \mathbb {S}$. Then $\bigl (x(t), \lambda (t)\bigr )$ converges weakly to a primal-dual solution of (1.1) as $t\rightarrow +\infty$.

### Proof

For proving this theorem, we make use of Opial’s Lemma (see Lemma A.5). Lemma 4.4 tells us that $\lim _{t\rightarrow +\infty }\left\| \bigl (x(t), \lambda (t)\bigr ) - (x_{*}, \lambda _{*})\right\|$ exists for every $(x_{*}, \lambda _{*}) \in \mathbb {S}$, which proves condition (i) of Opial’s Lemma. Now let $\bigl (\tilde{x}, \tilde{\lambda }\bigr )$ be any weak sequential cluster point of $\bigl ( x(t), \lambda (t)\bigr )$ as $t\rightarrow +\infty$, which means there exists a strictly increasing sequence $(t_{n})_{n\in \mathbb {N}} \subseteq [t_{0}, +\infty )$ such that

$$\begin{aligned} \bigl ( x(t_{n}), \lambda (t_{n})\bigr ) \rightharpoonup \bigl (\tilde{x}, \tilde{\lambda }\bigr ) \quad \text {as} \quad n \rightarrow +\infty . \end{aligned}$$

We want to show the remaining condition of Opial’s Lemma, which asks us to check that $\bigl (\tilde{x}, \tilde{\lambda }\bigr ) \in \mathbb {S}$. In other words, we must show that

4.24 $$\begin{aligned} \mathcal {L}\bigl (\tilde{x}, \lambda \bigr ) \leqslant \mathcal {L}\bigl (\tilde{x}, \tilde{\lambda }\bigr ) \leqslant \mathcal {L}\bigl (x, \tilde{\lambda }\bigr ) \quad \forall (x, \lambda ) \in \mathcal {X} \times \mathcal {Y}. \end{aligned}$$

Let $(x, \lambda ) \in \mathcal {X} \times \mathcal {Y}$ and $(x_{*}, \lambda _{*}) \in \mathbb {S}$ be fixed. Notice that the functions

$$\begin{aligned} f(\cdot ) + \bigl \langle \tilde{\lambda }, A(\cdot ) - Ax\bigr \rangle :\mathcal {X} \rightarrow \mathbb {R} \quad \text {and} \quad \bigl \langle \cdot , b - Ax\bigr \rangle :\mathcal {Y} \rightarrow \mathbb {R} \end{aligned}$$

are convex and continuous, therefore they are lower semicontinuous. According to a known result (see, for example, [14, Theorem 9.1]), they are also weakly lower semicontinuous. Therefore, we can derive that

$$\begin{aligned} \mathcal {L}\bigl (\tilde{x}, \tilde{\lambda }\bigr ) - \mathcal {L}\bigl (x, \tilde{\lambda }\bigr )&= f\bigl (\tilde{x}\bigr ) + \bigl \langle \tilde{\lambda }, A \tilde{x} - Ax\bigr \rangle - f(x) \\&\leqslant \liminf _{n\rightarrow +\infty } \Bigl [f(x(t_{n})) + \bigl \langle \tilde{\lambda }, Ax_{n} - Ax\bigr \rangle \Bigr ] - f(x) \\&= f(x_{*}) + \bigl \langle \tilde{\lambda }, b - Ax\bigr \rangle - f(x) \\&\leqslant f(x_{*}) - f(x) + \liminf _{n\rightarrow +\infty } \bigr \langle A^{*} \lambda (t_{n}), x_{*} - x\bigr \rangle \\&= f(x_{*}) - \bigl ( f(x) + \langle \lambda _{*}, Ax - b\rangle \bigr ) \\&= \mathcal {L}(x_{*}, \lambda _{*}) - \mathcal {L}(x, \lambda _{*}) \leqslant 0, \end{aligned}$$

where in the second and third equalities we used the fact that, as $n\rightarrow +\infty$, we have $f(x(t_{n})) \rightarrow f(x_{*})$ and $Ax(t_{n}) \rightarrow b$ (Theorem 3.3), and $A^{*}\lambda _{n} \rightarrow A^{*} \lambda _{*}$ (Theorem 4.8). Similarly, the weak lower semicontinuity of the function $\bigl \langle \lambda - \tilde{\lambda }, A(\cdot ) - b\bigr \rangle :\mathcal {X} \rightarrow \mathbb {R}$ yields

$$\begin{aligned} \mathcal {L}\bigl (\tilde{x}, \lambda \bigr ) - \mathcal {L}\bigl (\tilde{x}, \tilde{\lambda }\bigr ) = \bigl \langle \lambda - \tilde{\lambda }, A\tilde{x} - b\bigr \rangle \leqslant \liminf _{n\rightarrow +\infty } \bigl \langle \lambda - \tilde{\lambda }, Ax(t_{n}) - b\bigr \rangle = 0, \end{aligned}$$

We have thus showed (4.24) and the proof is concluded. $\square$

## Numerical Experiments

We will illustate the theoretical results by two numerical examples, with $\mathcal {X} = \mathbb {R}^{4}$ and $\mathcal {Y} = \mathbb {R}^{2}$. We will address two minimization problems with linear constraints; one with a strongly convex objective function and another with a convex objective function which is not strongly convex. In both cases, the linear constraints are dictated by

$$\begin{aligned} A = \begin{bmatrix} 1 &{} -1 &{} -1 &{} 0 \\ 0 &{} 1 &{} 0 &{} -1 \end{bmatrix} \qquad \text {and} \qquad b = \begin{bmatrix} 0 \\ 0 \end{bmatrix}. \end{aligned}$$

### Example 5.1

Consider the minimization problem

$$\begin{aligned} \begin{array}{rl} \min &{} f(x_{1}, x_{2}, x_{3}, x_{4}):= (x_{1} - 1)^{2} + (x_{2} - 1)^{2} + x_{3}^{2} + x_{4}^{2} \\ \text {subject to} &{} x_{1} - x_{2} - x_{3} = 0 \\ &{} x_{2} - x_{4} = 0. \end{array} \end{aligned}$$

The optimality conditions can be calculated and lead to the following primal-dual solution pair

$$\begin{aligned} x_{*} = \begin{bmatrix} 0.8 \\ 0.6 \\ 0.2 \\ 0.6 \end{bmatrix} \qquad \text {and} \qquad \lambda _{*} = \begin{bmatrix} 0.4 \\ 1.2 \end{bmatrix}. \end{aligned}$$

### Example 5.2

Consider the minimization problem

$$\begin{aligned} \begin{array}{rl} \min &{} f(x_{1}, x_{2}, x_{3}, x_{4}):= \log \left( 1 + e^{-x_{1} - x_{2}}\right) + x_{3}^{2} + x_{4}^{2} \\ \text {subject to} &{} x_{1} - x_{2} - x_{3} = 0 \\ &{} x_{2} - x_{4} = 0. \end{array} \end{aligned}$$

This problem is similar to the regularized logistic regression frequently used in machine learning. We cannot explicitly calculate the optimality conditions as in the previous case; instead, we use the last solution in the numerical experiment as the approximate solution.

To comply with Assumption 2, we choose $t_{0} > 0$, $\alpha = 8$, $\beta = 10$, $\theta = \frac{1}{6}$, and we test four different choices for the rescaling parameter: $\delta (t) = 1$ (i.e., the (PD-AVD) dynamics in [17, 45]), $\delta (t) = t$, $\delta (t) = t^{2}$ and $\delta (t) = t^{3}$. In both examples, the initial conditions are

$$\begin{aligned} x(t_{0}) = \begin{bmatrix} 0.5 \\ 0.5 \\ 0.5 \\ 0.5 \end{bmatrix}, \quad \lambda (t_{0}) = \begin{bmatrix} 0.2 \\ 0.2 \end{bmatrix}, \quad \dot{x}(t_{0}) = \begin{bmatrix} 0.5 \\ 0.5 \\ 0.5 \\ 0.5 \end{bmatrix} \quad \text {and} \quad \dot{\lambda }(t_{0}) = \begin{bmatrix} 0.5 \\ 0.5 \end{bmatrix}. \end{aligned}$$

For each choice of $\delta$, we plot, using a logarithmic scale, the primal-dual gap $\mathcal {L}\bigl (x(t), \lambda _{*}\bigr ) - \mathcal {L}\bigl (x_{*}, \lambda (t)\bigr )$, the feasibility measure $\left\Vert A x \left( t \right) - b \right\Vert$ and the functional values $\left|f \left( x \left( t \right) \right) - f_{*} \right|$, to highlight the theoretical result in Theorem 3.3. We also illustrate the findings from Theorem 4.8, namely, we plot the quantities $\left\Vert \nabla f \left( x \left( t \right) \right) - \nabla f \left( x_{*} \right) \right\Vert$ and $\left\Vert A^{*} \left( \lambda \left( t \right) - \lambda _{*} \right) \right\Vert$, as well as the velocity $\Vert ( \dot{x} (t), \dot{\lambda } (t) ) \Vert$.

**Fig. 1:**
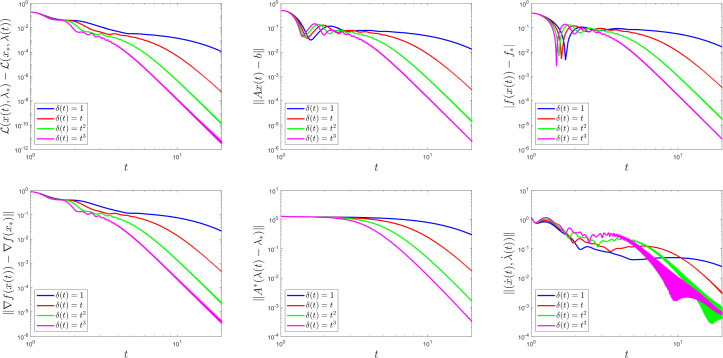
The function $\delta \left( t \right)$ influences convergence behaviour in Example 5.1

**Fig. 2:**
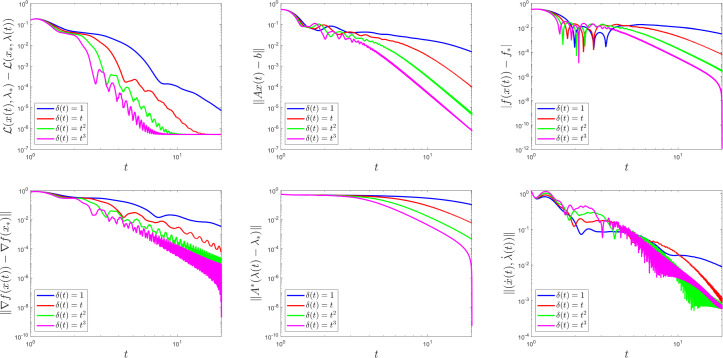
The function $\delta \left( t \right)$ influences convergence behaviour in Example 5.2

Figures 1 and 2 display these plots for Examples 5.1 and 5.2, respectively. As predicted by the theory, choosing faster-growing time rescaling parameters yields better convergence rates. This is not the case for the velocities.

**Fig. 3:**
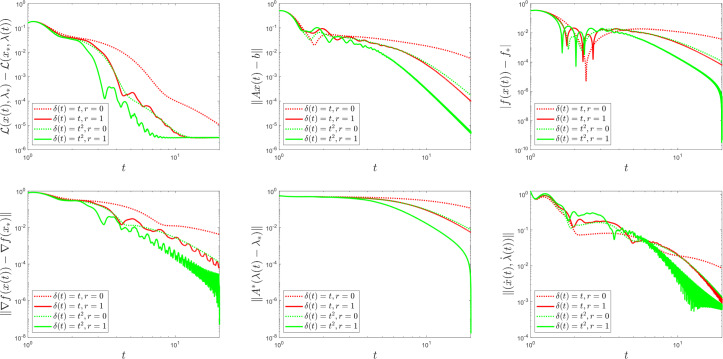
The function $\delta (t)$, as well as the parameter *r*, influence convergence behaviour in Example 5.2

Next we use Example 5.2 to compare the convergence behaviour of our system (2.6) with the one where the asymptotically vanishing damping term is chosen to be $\frac{\alpha }{t^{r}}$, for $r \in [0,1]$. Notice that $r = 1$ gives our system (2.6). When $r = 0$, in the setting of [30, Theorem 2.2], we know that the primal-dual gap exhibits a convergence rate of $\mathcal {O}\left( \frac{1}{t \delta (t)}\right)$ as $t\rightarrow +\infty$. This is illustrated in Fig. 3, were we plotted the combinations $\left( \delta (t) = t; r = 0 \right)$, $\left( \delta (t) = t; r = 1 \right)$, $\left( \delta (t) = t^{2}; r = 0 \right)$, and $\left( \delta (t) = t^{2}; r = 1 \right)$. In particular, observe that the rate predicted by [30, Theorem 2.2] for the primal-dual gap for the case $\left( \delta (t) = t^{2}; r = 0 \right)$ reads $\mathcal {O}\left( \frac{1}{t\cdot t^{2}}\right)$, while the rate predicted by our Theorem 3.3 for the case $\left( \delta (t) = t; r = 1 \right)$ reads $\mathcal {O}\left( \frac{1}{t^{2}\cdot t}\right)$. It is no surprise then to see the combinations $\left( \delta (t) = t^{2}; r = 0 \right)$ and $\left( \delta (t) = t; r = 1 \right)$ exhibiting similar convergence behaviour in Fig. 3.

**Fig. 4:**
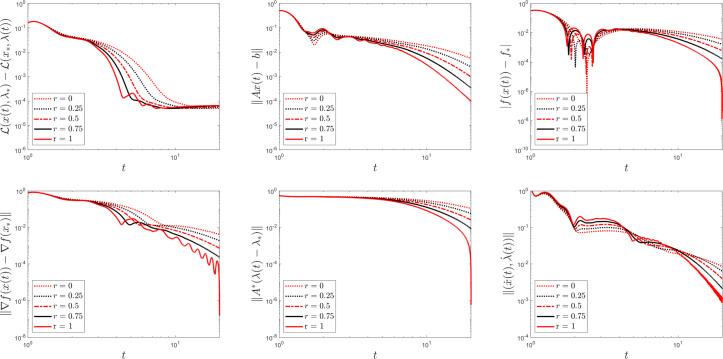
The parameter *r* influences convergence behaviour in Example 5.2

For better understanding, we run Example 5.2 once more to show the plots which result from fixing the time rescaling parameter $\delta (t) = t$ and varying $r \in \{0, 0.25, 0.5, 0.75, 1\}$. Notice how the convergence improves as *r* approaches 1. As $t\rightarrow +\infty$, [30, Theorem 2.7] predicts convergence rates of $\mathcal {O}\left( \frac{1}{t^{\tau } \delta (t)}\right)$ for the primal-dual gap and of $\mathcal {O}\left( \frac{1}{t^{\tau /2}}\right)$ for the velocities, which is reflected in our plots.

## Appendix

Here we collect the auxiliary results that are required to carry out many steps in out analysis.

A proof for the following lemma in the finite-dimensional case can be found in [28, Lemma 6]. The proof for the infinite-dimensional case is short and virtually identical, so we include it here for the sake of completeness.

### Lemma A.1

Assume that $t_{0} > 0$, $g: \left[ t_{0}, +\infty \right) \rightarrow \mathcal {Y}$ is a continuous differentiable function, $a: \left[ t_{0}, +\infty \right) \rightarrow [0, +\infty )$ is a continuous function, and $C \geqslant 0$. If, in the sense of Bochner integrability, we have

A.1 $$\begin{aligned} \left\| g(t) + \int _{t_{0}}^{t} a(s) g(s) ds\right\| \leqslant C \quad \forall t\geqslant t_{0} \end{aligned}$$

then

$$\begin{aligned} \sup _{t\geqslant t_{0}}\Vert g(t)\Vert \leqslant 2C < + \infty . \end{aligned}$$

### Proof

Define, for every $t\geqslant t_{0}$,

$$\begin{aligned} G(t):= e^{\int _{t_{0}}^{t} a(s) ds} \int _{t_{0}}^{t} a(s) g(s) ds. \end{aligned}$$

Fix $t\geqslant t_{0}$. The time derivative of *G* reads

$$\begin{aligned} \dot{G}(t)= & {} a(t) e^{\int _{t_{0}}^{t} a(s) ds} \int _{t_{0}}^{t} a(s) g(s) ds + e^{\int _{t_{0}}^{t} a(s) ds} a(t) g(t)\\= & {} a(t) e^{\int _{t_{0}}^{t} a(s) ds} \left[ g(t) + \int _{t_{0}}^{t} a(s) g(s) ds\right] , \end{aligned}$$

so by using (A.1) and the previous equality we arrive at

A.2 $$\begin{aligned} \bigl \Vert \dot{G}(t)\bigr \Vert \leqslant C a(t) e^{\int _{t_{0}}^{t} a(s) ds} = C \frac{d}{dt}\left( e^{\int _{t_{0}}^{t} a(s) ds}\right) . \end{aligned}$$

Since $G(t_{0}) = 0$, we have

$$\begin{aligned} G(t) = G(t) - G(t_{0}) = \int _{t_{0}}^{t} \dot{G}(s) ds, \end{aligned}$$

so by employing (A.2) and the previous equality we obtain, for every $t\geqslant t_{0}$,

$$\begin{aligned} e^{\int _{t_{0}}^{t} a(s) ds} \left\| \int _{t_{0}}^{t} a(s) g(s) ds\right\|&= \Vert G(t)\Vert \leqslant \int _{t_{0}}^{t}\bigl \Vert \dot{G}(s)\bigr \Vert ds \leqslant C \int _{t_{0}}^{t} \frac{d}{ds}\left( e^{\int _{t_{0}}^{s} a(\tau ) d\tau }\right) ds \\&\leqslant C \left[ e^{\int _{t_{0}}^{t} a(s) ds} - 1\right] \leqslant C e^{\int _{t_{0}}^{t} a(s) ds}. \end{aligned}$$

Dividing both sides of the previous inequality by $e^{\int _{t_{0}}^{t} a(s) ds}$ gives us

A.3 $$\begin{aligned} \left\| \int _{t_{0}}^{t} a(s) g(s) ds\right\| \leqslant C \quad \forall t\geqslant t_{0}. \end{aligned}$$

Now, by putting (A.1) and (A.3) we finally come to

$$\begin{aligned} \Vert g(t)\Vert \leqslant \left\| g(t) + \int _{t_{0}}^{t} a(s) g(s) ds\right\| + \left\| \int _{t_{0}}^{t} a(s) g(s) ds\right\| \leqslant 2C \quad \forall t\geqslant t_{0}, \end{aligned}$$

which leads to the announced statement. $\square$

The proofs for the following results can be found in [17, Lemma A.1] and [1, Lemma 5.2], respectively.

### Lemma A.2

Let $0 < t_{0} \leqslant r \leqslant +\infty$ and $h:\left[ t_{0}, +\infty \right) \rightarrow [0, +\infty )$ be a continuous function. For every $\alpha > 1$ it holds

$$\begin{aligned} \int _{t_{0}}^{r} \frac{1}{t^{\alpha }} \left[ \int _{t_{0}}^{t}s^{\alpha - 1} h(s)\right] dt \leqslant \frac{1}{\alpha - 1} \int _{t_{0}}^{r} h(t) dt. \end{aligned}$$

If $r = +\infty$, then equality holds.

### Lemma A.3

Let $t_{0} > 0$, $1 \leqslant p < +\infty$ and $1\leqslant q \leqslant +\infty$. Suppose that $F \in \mathbb {L}^{p}\left[ t_{0}, +\infty \right)$ is a locally absolutely continuous nonnegative function, $G \in \mathbb {L}^{q}\left[ t_{0}, +\infty \right)$ and

$$\begin{aligned} \dot{F}(t) \leqslant G(t) \qquad \forall t\geqslant t_{0}. \end{aligned}$$

Then, $$\lim _{t\rightarrow +\infty } F(t) = 0$$.

The following lemma is a slight variation of results already present in the literature. See, for example, [5, Lemma A.2].

### Lemma A.4

Let $t_{0} > 0$, $\alpha > 1$, and let $\phi : [t_{0}, +\infty ) \rightarrow \mathbb {R}$ be a twice continuously differentiable function bounded from below. Furthermore, assume $w: [t_{0}, +\infty ) \rightarrow [0, +\infty )$ to be a continuously differentiable function such that $t\mapsto t w(t)$ belongs to $\mathbb {L}^{1}[t_{0}, +\infty )$ and

$$\begin{aligned} t\ddot{\phi }(t) + \alpha \dot{\phi }(t) + t^{2}\dot{w}(t) \leqslant 0 \qquad \forall t\geqslant t_{0}. \end{aligned}$$

Then, the positive part $[\dot{\phi }]_{+}$ of $\dot{\phi }$ belongs to $\mathbb {L}^{1}[t_{0}, +\infty )$ and the limit $\lim _{t\rightarrow +\infty } \phi (t)$ is a real number.

### Proof

Fix $t\geqslant t_{0}$. Adding $(\alpha + 1)t w(t)$ to both sides of the previous inequality and then multiplying it by $t^{\alpha - 1}$ yields

$$\begin{aligned} \frac{d}{dt}\bigl (t^{\alpha } \dot{\phi }(t)\bigr ) + \frac{d}{dt}\bigl (t^{\alpha + 1}w(t)\bigr ) \leqslant (\alpha + 1)t^{\alpha }w(t). \end{aligned}$$

Since the previous inequality holds for any $t\geqslant t_{0}$, we can integrate it from $t_{0}$ to $t\geqslant t_{0}$ to get

$$\begin{aligned} t^{\alpha } \dot{\phi }(t) - t_{0}^{\alpha } \dot{\phi }(t_{0}) + t^{\alpha + 1}w(t) - t_{0}^{\alpha + 1}w(t_{0}) \leqslant (\alpha + 1) \int _{t_{0}}^{t} s^{\alpha }w(s) ds. \end{aligned}$$

After dropping the nonnegative term $t^{\alpha + 1}w(t)$ and dividing by $t^{\alpha }$ we arrive at

$$\begin{aligned} \dot{\phi }(t) \leqslant \frac{\widetilde{C}}{t^{\alpha }} + \frac{\alpha + 1}{t^{\alpha }} \int _{t_{0}}^{t}s^{\alpha } w(s) ds \qquad \forall t\geqslant t_{0}, \end{aligned}$$

where

$$\begin{aligned} \widetilde{C}:= t_{0}^{\alpha } \bigl | \dot{\phi }(t_{0})\bigr | + t_{0}^{\alpha + 1}w(t_{0}), \end{aligned}$$

which further leads to

$$\begin{aligned}{}[\dot{\phi }(t)]_{+} \leqslant \frac{\widetilde{C}}{t^{\alpha }} + \frac{\alpha + 1}{t^{\alpha }} \int _{t_{0}}^{t}s^{\alpha }w(s) ds \qquad \forall t\geqslant t_{0}. \end{aligned}$$

Now, we integrate this inequality from $t_{0}$ to $r\geqslant t_{0}$ and we apply Lemma A.2 with $h: [t_{0}, +\infty ) \rightarrow [0, +\infty )$ given by $h(s):= sw(s)$ to obtain

$$\begin{aligned} \int _{t_{0}}^{r}[\dot{\phi }(t)]_{+}dt&\leqslant \widetilde{C}\int _{t_{0}}^{r} \frac{1}{t^{\alpha }}dt + (\alpha + 1) \int _{t_{0}}^{r}\frac{1}{t^{\alpha }} \left[ \int _{t_{0}}^{t} s^{\alpha - 1}\cdot sw(s)ds\right] dt \\&\leqslant \frac{\widetilde{C}}{1 - \alpha }\left( \frac{1}{t_{0}^{\alpha - 1}} - \frac{1}{r^{\alpha - 1}}\right) + \frac{\alpha + 1}{1 - \alpha } \int _{t_{0}}^{r}tw(t) dt. \end{aligned}$$

By hypothesis, as $r\rightarrow +\infty$, the right hand side of the previous inequality is finite. In other words,

$$\begin{aligned} \int _{t_{0}}^{+\infty }[\dot{\phi }(t)]_{+}dt < +\infty . \end{aligned}$$

The previous statement, together with the fact that we assumed that $\phi$ was bounded from below, allow us to deduce that the function $\psi : [t_{0}, +\infty ) \rightarrow \mathbb {R}$ given by

$$\begin{aligned} \psi (t):= \phi (t) - \int _{t_{0}}^{t}[\dot{\phi }(s)]_{+}ds \end{aligned}$$

is also bounded from below. An easy computation shows that $\dot{\psi }$ is nonpositive on $[t_{0}, +\infty )$, thus $\psi$ is nonincreasing on $[t_{0}, +\infty )$. These facts imply that $\lim _{t\rightarrow +\infty }\psi (t)$ is a real number. Finally, we conclude that

$$\begin{aligned} \lim _{t\rightarrow +\infty } \phi (t) = \lim _{t\rightarrow +\infty }\psi (t) + \int _{t_{0}}^{+\infty }[\dot{\phi }(s)]_{+}ds \, \in \, \mathbb {R}. \end{aligned}$$

$$\square $$

The proof for Opial’s Lemma can be found in [36].

### Lemma A.5

(Opial’s Lemma) Let $\mathcal {H}$ be a real Hilbert space, $S \subseteq \mathcal {H}$ a nonempty set, $t_{0} > 0$ and $z: \left[ t_{0}, +\infty \right) \rightarrow \mathcal {H}$ a mapping that satisfies

(i) for every $z_{*} \in S$, $\lim _{t\rightarrow +\infty } \Vert z(t) - z_{*}\Vert$ exists;
(ii) every weak sequential cluster point of the trajectory *z*(*t*) as $t \rightarrow +\infty$ belongs to *S*.

Then, *z*(*t*) converges weakly to an element of *S* as $t \rightarrow +\infty$.

## Appendix B: Missing Proofs

### Proof of Lemma 3.1

Let $t \geqslant t_{0}$ be fixed. Since $x_{*} \in \mathbb {F}$, we have

$$\begin{aligned} \nabla _{x} \left( \mathcal {L}_{\beta }\left( x \left( t \right) , \lambda _{*} \right) - \mathcal {L}_{\beta }\left( x_{*}, \lambda \left( t \right) \right) \right)&= \nabla _{x} \mathcal {L}_{\beta }\left( x \left( t \right) , \lambda _{*} \right) \\&= \nabla f \left( x \left( t \right) \right) + A^{*} \lambda _{*} + \beta A^{*} \left( Ax \left( t \right) - b \right) , \\ \nabla _{\lambda } \left( \mathcal {L}_{\beta }\left( x \left( t \right) , \lambda _{*} \right) - \mathcal {L}_{\beta }\left( x_{*}, \lambda \left( t \right) \right) \right)&= - \nabla _{\lambda } \mathcal {L}_{\beta }\left( x_{*}, \lambda \left( t \right) \right) = 0 . \end{aligned}$$

Under these expressions, the system (2.6) can be equivalently written as

$$\begin{aligned} \left( \ddot{x} \left( t \right) , \ddot{\lambda } \left( t \right) \right)&= - \dfrac{\alpha }{t} \left( \dot{x} \left( t \right) , \dot{\lambda } \left( t \right) \right) - \delta \left( t \right) \bigl ( \nabla _{x} \mathcal {L}_{\beta }\left( x \left( t \right) , \lambda _{*} \right) , 0 \bigr ) \\&\quad - \delta (t)\left( A^{*} \left( \lambda \left( t \right) - \lambda _{*} + \theta t \dot{\lambda } \left( t \right) \right) , - \Bigl ( A \left( x \left( t \right) + \theta t \dot{x} \left( t \right) \right) - b \Bigr )\right) , \end{aligned}$$

which leads to

$$\begin{aligned} \dot{v} \left( t \right)&= (1 + \theta )\left( \dot{x} \left( t \right) , \dot{\lambda } \left( t \right) \right) + \theta t \left( \ddot{x} \left( t \right) , \ddot{\lambda } \left( t \right) \right) \\&= - \xi \left( \dot{x} \left( t \right) , \dot{\lambda } \left( t \right) \right) - \theta t \delta \left( t \right) \left( t \right) \bigl ( \nabla _{x} \mathcal {L}_{\beta }\left( x \left( t \right) , \lambda _{*} \right) , 0 \bigr ) \\&\quad - \theta t \delta \left( t \right) \begin{pmatrix} A^{*} \left( \lambda \left( t \right) - \lambda _{*} + \theta t \dot{\lambda } \left( t \right) \right) , - \Bigl ( A \left( x \left( t \right) + \theta t \dot{x} \left( t \right) \right) - b \Bigr ) \end{pmatrix}. \end{aligned}$$

We get from the distributive property of the inner product

$$\begin{aligned}&\left\langle v \left( t \right) , \dot{v} \left( t \right) \right\rangle \\&\quad = - \xi \left\langle \bigl ( x \left( t \right) , \lambda \left( t \right) \bigr ) - (x_{*}, \lambda _{*}) , \left( \dot{x} \left( t \right) , \dot{\lambda } \left( t \right) \right) \right\rangle - \xi \theta t \left\Vert \left( \dot{x} \left( t \right) , \dot{\lambda } \left( t \right) \right) \right\Vert ^{2} \\&\qquad - \theta t \delta \left( t \right) \left\langle \left( \nabla _{x} \mathcal {L}_{\beta }\left( x \left( t \right) , \lambda _{*} \right) , 0 \right) , \bigl ( x \left( t \right) , \lambda \left( t \right) \bigr ) - (x_{*}, \lambda _{*}) \right\rangle \\&\qquad - \theta ^{2} t^{2} \delta \left( t \right) \left\langle \left( \nabla _{x} \mathcal {L}_{\beta }\left( x \left( t \right) , \lambda _{*} \right) , 0 \right) , \left( \dot{x} \left( t \right) , \dot{\lambda } \left( t \right) \right) \right\rangle \\&\qquad - \theta t \delta \left( t \right) \left\langle \lambda \left( t \right) - \lambda _{*} + \theta t \dot{\lambda } \left( t \right) , Ax \left( t \right) - Ax_{*} \right\rangle \\&\qquad - \theta ^{2} t^{2} \delta \left( t \right) \left\langle \lambda \left( t \right) - \lambda _{*} + \theta t \dot{\lambda } \left( t \right) , A \dot{x} \left( t \right) \right\rangle \\&\qquad + \theta t \delta \left( t \right) \left\langle A \left( x \left( t \right) + \theta t \dot{x} \left( t \right) \right) - b , \lambda \left( t \right) - \lambda _{*} \right\rangle \\&\qquad + \theta ^{2} t^{2} \delta \left( t \right) \left\langle A \left( x \left( t \right) + \theta t \dot{x} \left( t \right) \right) - b , \dot{\lambda } \left( t \right) \right\rangle . \end{aligned}$$

Since $x_{*} \in \mathbb {F}$, the last four terms in the above identity vanish. Indeed,

$$\begin{aligned}&- \left\langle \lambda \left( t \right) - \lambda _{*} + \theta t \dot{\lambda } \left( t \right) , Ax \left( t \right) - Ax_{*} \right\rangle - \theta t \left\langle \lambda \left( t \right) - \lambda _{*} + \theta t \dot{\lambda } \left( t \right) , A \dot{x} \left( t \right) \right\rangle \\&\quad + \left\langle A \left( x \left( t \right) + \theta t \dot{x} \left( t \right) \right) - b , \lambda \left( t \right) - \lambda _{*} \right\rangle + \theta t \left\langle A \left( x \left( t \right) + \theta t \dot{x} \left( t \right) \right) - b , \dot{\lambda } \left( t \right) \right\rangle \\&= - \left\langle \lambda \left( t \right) - \lambda _{*} + \theta t \dot{\lambda } \left( t \right) , Ax \left( t \right) - b \right\rangle - \theta t \left\langle \lambda \left( t \right) - \lambda _{*} + \theta t \dot{\lambda } \left( t \right) , A \dot{x} \left( t \right) \right\rangle \\&\quad + \left\langle Ax \left( t \right) - b , \lambda \left( t \right) - \lambda _{*} \right\rangle + \theta t \left\langle A \dot{x} \left( t \right) , \lambda \left( t \right) - \lambda _{*} \right\rangle \\&\quad + \theta t \left\langle Ax \left( t \right) - b , \dot{\lambda } \left( t \right) \right\rangle + \theta ^{2} t^{2} \left\langle A \dot{x} \left( t \right) , \dot{\lambda } \left( t \right) \right\rangle \\&= 0 . \end{aligned}$$

Therefore, differentiating $\mathcal {E}$ with respect to *t* gives

B.1 $$\begin{aligned} \dfrac{d}{dt} \mathcal {E}\left( t \right)&= \theta ^{2} t \left( 2 \delta \left( t \right) + t \dot{\delta } \left( t \right) \right) \left( \mathcal {L}_{\beta }\left( x \left( t \right) , \lambda _{*} \right) - \mathcal {L}_{\beta }\left( x_{*} , \lambda \left( t \right) \right) \right) \nonumber \\&\quad + \theta ^{2} t^{2} \delta (t) \left\langle \left( \nabla _{x} \mathcal {L}_{\beta }\left( x \left( t \right) , \lambda _{*} \right) , 0 \right) , \left( \dot{x} \left( t \right) , \dot{\lambda } \left( t \right) \right) \right\rangle \nonumber \\&\quad + \left\langle v \left( t \right) , \dot{v} \left( t \right) \right\rangle + \xi \left\langle \bigl ( x \left( t \right) , \lambda \left( t \right) \bigr ) - (x_{*}, \lambda _{*}) , \left( \dot{x} \left( t \right) , \dot{\lambda } \left( t \right) \right) \right\rangle \nonumber \\&= \theta ^{2} t \left( 2 \delta \left( t \right) + t \dot{\delta } \left( t \right) \right) \left( \mathcal {L}_{\beta }\left( x \left( t \right) , \lambda _{*} \right) - \mathcal {L}_{\beta }\left( x_{*} , \lambda \left( t \right) \right) \right) - \xi \theta t \left\Vert \left( \dot{x} \left( t \right) , \dot{\lambda } \left( t \right) \right) \right\Vert ^{2} \nonumber \\&\quad - \theta t \delta (t) \left\langle \left( \nabla _{x} \mathcal {L}_{\beta }\left( x \left( t \right) , \lambda _{*} \right) , 0 \right) , \bigl ( x \left( t \right) , \lambda \left( t \right) \bigr ) - (x_{*}, \lambda _{*}) \right\rangle . \end{aligned}$$

Furthermore, the convexity of *f* and the fact that $x_{*} \in \mathbb {F}$ guarantee

B.2 $$\begin{aligned}&- \left\langle \left( \nabla _{x} \mathcal {L}_{\beta }\left( x \left( t \right) , \lambda _{*} \right) , 0 \right) , \bigl ( x \left( t \right) , \lambda \left( t \right) \bigr ) - (x_{*}, \lambda _{*}) \right\rangle \nonumber \\&\quad = \left\langle \nabla f \left( x \left( t \right) \right) , x_{*} - x \left( t \right) \right\rangle + \left\langle A^{*} \lambda _{*} , x_{*} - x \left( t \right) \right\rangle + \beta \left\langle A^{*} \left( Ax \left( t \right) - b \right) , x_{*} - x \left( t \right) \right\rangle \nonumber \\&\quad \leqslant - \left( f \left( x \left( t \right) \right) - f \left( x_{*} \right) \right) - \left\langle \lambda _{*} , Ax \left( t \right) - b \right\rangle - \beta \left\Vert Ax \left( t \right) - b \right\Vert ^{2} \nonumber \\&\quad = - \left( \mathcal {L}_{\beta }\left( x \left( t \right) , \lambda _{*} \right) - \mathcal {L}_{\beta }\left( x_{*} , \lambda \left( t \right) \right) \right) - \dfrac{\beta }{2} \left\Vert Ax \left( t \right) - b \right\Vert ^{2} , \end{aligned}$$

where we recall that the second equality comes from (2.5). By multiplying this inequality by $\theta t \delta (t)$ and combining it with (B.1), the coefficient attached to the primal-dual gap becomes

$$\begin{aligned} \theta ^{2} t \left( 2\delta (t) + t \dot{\delta }(t)\right) - \theta t \delta (t) = - \theta ^{2} t \left( \frac{1 - 2\theta }{\theta } \delta (t) - t\dot{\delta }(t)\right) = - \theta ^{2} t \sigma (t), \end{aligned}$$

which finally gives the desired statement. $\square$

### Proof of Theorem 3.2

(i) Recall that Assumption 1 implies $\sigma (t) \geqslant 0$ for all $t\geqslant t_{0}$ and $\xi \geqslant 0$. Moreover, $(x_{*}, \lambda _{*}) \in \mathbb {S}$ yields $x_{*} \in \mathbb {F}$. Therefore, we can apply (3.7) and Lemma 3.1 to obtain, for every $t\geqslant t_{0}$,

B.3 $$\begin{aligned} \frac{d}{dt}\mathcal {E}(t)&\leqslant -\theta ^{2} t \sigma (t) \left( \mathcal {L}_{\beta }\left( x \left( t \right) , \lambda _{*} \right) - \mathcal {L}_{\beta }\left( x_{*} , \lambda \left( t \right) \right) \right) \nonumber \\&\quad - \frac{1}{2}\beta \theta t \delta (t) \left\Vert Ax \left( t \right) - b \right\Vert ^{2} - \xi \theta t \left\Vert \left( \dot{x} \left( t \right) , \dot{\lambda } \left( t \right) \right) \right\Vert ^{2} \leqslant 0. \end{aligned}$$

This means that $\mathcal {E}$ is nonincreasing on $\left[ t_{0}, +\infty \right)$. For every $t\geqslant t_{0}$, by integrating (B.3) from $t_{0}$ to *t*, we obtain

$$\begin{aligned}&\theta ^{2}\int _{t_{0}}^{t} t\sigma (t)\Bigl (\mathcal {L}(x(s), \lambda _{*}) - \mathcal {L}(x_{*}, \lambda (s))\Bigr ) ds \\&\quad + \frac{\beta \theta }{2}\int _{t_{0}}^{t}s \delta (s)\Vert Ax(s) - b\Vert ^{2}ds + \xi \theta \int _{t_{0}}^{t}s\left\| \left( \dot{x}(s), \dot{\lambda }(s)\right) \right\| ^{2} ds \\&\leqslant \mathcal {E}(t_{0}) - \mathcal {E}(t) \leqslant \mathcal {E}(t_{0}), \end{aligned}$$

where the last inequality follows from (3.8). Since all quantities inside the integrals are nonnegative, we obtain (3.10)–(3.12) by letting $t \rightarrow +\infty$.

(ii) Let $t \geqslant t_{0}$ be fixed. Inequality (B.3) tells us that $\mathcal {E}$ is nonincreasing on $\left[ t_{0}, +\infty \right)$.

B.4 $$\begin{aligned} \begin{aligned}&\theta ^{2} t^{2} \delta (t) \Bigl (\mathcal {L}_{\beta }\left( x(t), \lambda _{*} \right) - \mathcal {L}_{\beta }(x_{*}, \lambda (t))\Bigr ) \\&\quad + \left\| v(t)\right\| ^{2} + \frac{\xi }{2}\left\| \bigl (x(t), \lambda (t)\bigr ) - (x_{*}, \lambda _{*})\right\| ^{2} \leqslant \mathcal {E}(t_{0}). \end{aligned} \end{aligned}$$

Assuming $\alpha > 3$ and $\frac{1}{2} \geqslant \theta > \frac{1}{\alpha - 1}$, we immediately see $\xi > 0$. From (B.4) we obtain

B.5 $$\begin{aligned} \left\| \bigl (x(t), \lambda (t)\bigr ) - (x_{*}, \lambda _{*})\right\| ^{2} \leqslant \frac{2\mathcal {E}(t_{0})}{\xi }, \end{aligned}$$

and

B.6 $$\begin{aligned} \left\| v(t)\right\| = \left\| \bigl (x(t), \lambda (t)\bigr ) - (x_{*}, \lambda _{*}) + \theta t \left( \dot{x}(t), \dot{\lambda }(t)\right) \right\| \leqslant \sqrt{2\mathcal {E}(t_{0})}. \end{aligned}$$

The estimate (B.5) leads to the boundedness of the trajectory. Moreover, applying the triangle inequality and (B.5)–(B.6), we obtain

B.7 $$\begin{aligned} t\left\Vert \left( \dot{x} \left( t \right) , \dot{\lambda } \left( t \right) \right) \right\Vert&\leqslant \frac{1}{\theta } \left( \left\| \bigl (x(t), \lambda (t)\bigr ) - (x_{*}, \lambda _{*})\right\| + \left\| v {(t)} \right\| \right) \nonumber \\&\leqslant \frac{1}{\theta }\left( \sqrt{\frac{2\mathcal {E}(t_{0})}{\xi }} + \sqrt{2\mathcal {E}(t_{0})}\right) = \frac{1}{\theta }\left( \frac{1}{\sqrt{\xi }} + 1\right) \sqrt{2\mathcal {E}(t_{0})}, \end{aligned}$$

which gives the desired convergence rate. $\square$

### Proof of Proposition 4.2

Thanks to $\nabla f$ being $\ell$-Lipschitz continuous, we can use (1.3) to refine relation (B.2) in the proof of Lemma 3.1

$$\begin{aligned}&- \left\langle \left( \nabla _{x} \mathcal {L}_{\beta }\left( x \left( t \right) , \lambda _{*} \right) , 0 \right) , \bigl ( x \left( t \right) , \lambda \left( t \right) \bigr ) - (x_{*}, \lambda _{*}) \right\rangle \\&\quad = \langle \nabla f(x(t)), x_{*} - x(t)\rangle + \left\langle A^{*}\lambda _{*}, x_{*} - x(t)\right\rangle + \beta \left\langle A^{*}(Ax(t) - b), x_{*} - x(t)\right\rangle \\&\quad \leqslant - (f(x(t)) - f(x_{*})) - \frac{1}{2\ell }\left\| \nabla f(x(t)) - \nabla f(x_{*})\right\| ^{2} - \langle \lambda _{*}, Ax(t) - b\rangle \\&\qquad - \beta \left\Vert Ax \left( t \right) - b \right\Vert ^{2} \\&\quad = - \left( \mathcal {L}_{\beta }\left( x \left( t \right) , \lambda _{*} \right) - \mathcal {L}_{\beta }\left( x_{*} , \lambda \left( t \right) \right) \right) - \frac{1}{2\ell } \left\| \nabla f(x(t)) - \nabla f(x_{*})\right\| ^{2} \\&\qquad - \frac{\beta }{2}\left\Vert Ax \left( t \right) - b \right\Vert ^{2}. \end{aligned}$$

Consequently, combining this inequality with (B.1) yields, for every $t\geqslant t_{0}$

$$\begin{aligned} \frac{d}{dt}\mathcal {E}(t)&\leqslant - \theta ^{2} t \sigma (t) \left( \mathcal {L}_{\beta }\left( x \left( t \right) , \lambda _{*} \right) - \mathcal {L}_{\beta }\left( x_{*} , \lambda \left( t \right) \right) \right) - \xi \theta t \left\Vert \left( \dot{x} \left( t \right) , \dot{\lambda } \left( t \right) \right) \right\Vert ^{2} \\&\quad - \frac{\theta t\delta (t)}{2\ell }\left\Vert \nabla f \left( x \left( t \right) \right) - \nabla f \left( x_{*} \right) \right\Vert ^{2} - \frac{\theta \beta t \delta (t)}{2}\left\Vert Ax \left( t \right) - b \right\Vert ^{2} \\&\leqslant - \frac{\theta t \delta (t)}{2\ell } \left\Vert \nabla f \left( x \left( t \right) \right) - \nabla f \left( x_{*} \right) \right\Vert ^{2}. \end{aligned}$$

Integration of this inequality produces (4.4).

The finiteness of the second integral is only entailed by (3.11) when $\beta > 0$. For the general case $\beta \geqslant 0$, we use (3.14) and the fact that $\delta$ is nondecreasing on $\left[ t_{0}, +\infty \right)$ to obtain

$$\begin{aligned} \int _{t_{0}}^{+ \infty } t\delta (t) \left\Vert Ax \left( t \right) - b \right\Vert ^{2} dt \leqslant 4 C_{1}^{2} \int _{t_{0}}^{+ \infty } \frac{1}{t^{3} \delta (t)} dt \leqslant \frac{4 C_{1}^{2}}{\delta (t_{0})} \int _{t_{0}}^{+ \infty } \dfrac{1}{t^{3}} dt < + \infty , \end{aligned}$$

and the proof is complete. $\square$

### Proof of Lemma 4.3

Let $t\geqslant t_{0}$ be fixed. Differentiating *W* with respect to time yields

$$\begin{aligned} \dot{W}(t)&= \dot{\delta }(t)\Bigl [\mathcal {L}_{\beta }\left( x(t), \lambda _{*} \right) - \mathcal {L}_{\beta }(x_{*}, \lambda (t))\Bigr ] \\&\quad + \delta (t) \Bigl [\left\langle \nabla _{x}\mathcal {L}_{\beta }\left( x(t), \lambda _{*} \right) , \dot{x}(t)\right\rangle - \left\langle \nabla _{\lambda }\mathcal {L}_{\beta }(x_{*}, \lambda (t)), \dot{\lambda }(t)\right\rangle \Bigr ] \\&\quad + \langle \ddot{x}(t), \dot{x}(t)\rangle + \langle \ddot{\lambda }(t), \dot{\lambda }(t)\rangle . \end{aligned}$$

Recall the formulas for the gradients of $\mathcal {L}$

$$\begin{aligned} \nabla _{x}\mathcal {L}_{\beta }\left( x(t), \lambda _{*} \right)&= \nabla f(x(t)) + A^{*}\lambda _{*} + \beta A^{*}(Ax(t) - b), \\ \nabla _{\lambda }\mathcal {L}_{\beta }(x_{*}, \lambda (t))&= Ax_{*} - b = 0, \end{aligned}$$

since $x_{*} \in \mathbb {F}$. Plugging this into the expression for $\dot{W}(t)$ gives us

$$\begin{aligned} \dot{W}(t)&= \dot{\delta }(t)\Bigl [\mathcal {L}_{\beta }\left( x(t), \lambda _{*} \right) - \mathcal {L}_{\beta }(x_{*}, \lambda (t))\Bigr ] \\&\quad + \delta (t) \left\langle \nabla _{x}\mathcal {L}_{\beta }(x(t), \lambda (t) + \theta t \dot{\lambda }(t)), \dot{x}(t)\right\rangle + \langle \ddot{x}(t), \dot{x}(t)\rangle \\&\quad - \delta (t) \left\langle \lambda (t) - \lambda _{*} + \theta t \dot{\lambda }(t), A\dot{x}(t)\right\rangle \\&\quad - \delta (t) \left\langle \nabla _{\lambda }\mathcal {L}_{\beta }(x(t) + \theta t \dot{x}(t), \lambda (t)), \dot{\lambda (t)}\right\rangle + \langle \ddot{\lambda }(t), \dot{\lambda }(t)\rangle \\&\quad + \delta (t) \left\langle Ax(t) - b + \theta t A\dot{x}(t), \dot{\lambda }(t)\right\rangle . \end{aligned}$$

By regrouping and using (2.6), we arrive at

B.8 $$\begin{aligned} \begin{aligned} \dot{W}(t)&= \dot{\delta }(t)\Bigl [\mathcal {L}_{\beta }\left( x(t), \lambda _{*} \right) - \mathcal {L}_{\beta }(x_{*}, \lambda (t))\Bigr ] - \frac{\alpha }{t}\left\Vert \dot{x} \left( t \right) \right\Vert ^{2} - \frac{\alpha }{t}\Vert \dot{\lambda }(t)\Vert ^{2} \\&- \delta (t) \left\langle \lambda (t) - \lambda _{*}, A\dot{x}(t)\right\rangle + \delta (t) \bigl \langle Ax(t) - b, \dot{\lambda }(t)\bigr \rangle . \end{aligned} \end{aligned}$$

On the other hand, by the chain rule, we have

$$\begin{aligned} \dot{\varphi }(t)&= \langle x(t) - x_{*}, \dot{x}(t)\rangle + \bigl \langle \lambda (t) - \lambda _{*}, \dot{\lambda }(t)\bigr \rangle , \\ \ddot{\varphi }(t)&= \langle x(t) - x_{*}, \ddot{x}(t)\rangle + \left\Vert \dot{x} \left( t \right) \right\Vert ^{2} + \bigl \langle \lambda (t) - \lambda _{*}, \ddot{\lambda }(t)\bigr \rangle + \bigl \Vert \dot{\lambda }(t)\bigr \Vert ^{2}. \end{aligned}$$

By combining these relations, (2.6) and the fact that $x_{*} \in \mathbb {F}$, we get

B.9 $$\begin{aligned} \ddot{\varphi }(t) + \frac{\alpha }{t}\dot{\varphi }(t)&= \left\langle x(t) - x_{*}, \ddot{x}(t) + \frac{\alpha }{t}\dot{x}(t)\right\rangle + \left\langle \lambda (t) - \lambda _{*}, \ddot{\lambda }(t) + \frac{\alpha }{t}\dot{\lambda }(t)\right\rangle + \left\Vert \dot{x} \left( t \right) \right\Vert ^{2} \nonumber \\&\quad + \left\Vert \dot{\lambda } \left( t \right) \right\Vert ^{2} \nonumber \\&= - \bigl \langle x(t) - x_{*}, \delta (t) \nabla _{x}\mathcal {L}_{\beta }(x(t), \lambda (t) + \theta t \dot{\lambda }(t))\bigr \rangle \nonumber \\&\quad + \bigl \langle \lambda (t) - \lambda _{*}, \delta (t) \nabla _{\lambda }\mathcal {L}_{\beta }(x(t) + \theta t \dot{\lambda }(t), \lambda (t))\bigr \rangle + \left\Vert \dot{x} \left( t \right) \right\Vert ^{2} + \left\Vert \dot{\lambda } \left( t \right) \right\Vert ^{2} \nonumber \\&= -\bigl \langle x(t) - x_{*}, \delta (t) \nabla _{x} \mathcal {L}_{\beta }\left( x(t), \lambda _{*} \right) \bigr \rangle \nonumber \\&\quad - \bigl \langle Ax(t) - b, \delta (t) \bigl (\lambda (t) - \lambda _{*} + \theta t \dot{\lambda }(t)\bigr )\bigr \rangle \nonumber \\&\quad + \bigl \langle \lambda (t) - \lambda _{*}, \delta (t) \bigl (Ax(t) - b + \theta t A\dot{x}(t)\bigr )\bigr \rangle + \left\Vert \dot{x} \left( t \right) \right\Vert ^{2} + \left\Vert \dot{\lambda } \left( t \right) \right\Vert ^{2} \nonumber \\&= -\delta (t) \bigl \langle x(t) - x_{*}, \nabla _{x} \mathcal {L}_{\beta }\left( x(t), \lambda _{*} \right) \bigr \rangle -\theta t \delta (t) \bigl \langle Ax(t) - b, \dot{\lambda }(t)\bigr \rangle \nonumber \\&\quad + \theta t \delta (t) \bigl \langle \lambda (t) - \lambda _{*}, A \dot{x}(t)\bigr \rangle + \left\Vert \dot{x} \left( t \right) \right\Vert ^{2} + \left\Vert \dot{\lambda } \left( t \right) \right\Vert ^{2} \end{aligned}$$

The Lipschitz continuity of $\nabla f$ entails

$$\begin{aligned}&-\left\langle x(t) - x_{*}, \nabla _{x} \mathcal {L}_{\beta }\left( x(t), \lambda _{*} \right) \right\rangle \\&\quad = -\langle x(t) - x_{*}, \nabla f(x(t))\rangle - \bigl \langle x(t) - x_{*}, A^{*}\lambda _{*}\bigr \rangle - \beta \left\Vert Ax \left( t \right) - b \right\Vert ^{2} \\&\quad \leqslant -(f(x(t)) - f(x_{*})) - \frac{1}{2\ell } \left\Vert \nabla f \left( x \left( t \right) \right) - \nabla f \left( x_{*} \right) \right\Vert ^{2}\\&\qquad - \langle \lambda _{*}, Ax(t) - b\rangle - \beta \left\Vert Ax \left( t \right) - b \right\Vert ^{2} \\&\quad = -\Bigl ( \mathcal {L}_{\beta }\left( x(t), \lambda _{*} \right) - \mathcal {L}_{\beta }(x_{*}, \lambda (t))\Bigr ) - \frac{1}{2\ell }\left\Vert \nabla f \left( x \left( t \right) \right) - \nabla f \left( x_{*} \right) \right\Vert ^{2}\\&\qquad - \frac{\beta }{2}\left\Vert Ax \left( t \right) - b \right\Vert ^{2}. \end{aligned}$$

This, together with (B.9), implies

B.10 $$\begin{aligned} \ddot{\varphi }(t) + \frac{\alpha }{t}\dot{\varphi }(t)&\leqslant - \delta (t)\Bigl ( \mathcal {L}_{\beta }\left( x(t), \lambda _{*} \right) - \mathcal {L}_{\beta }(x_{*}, \lambda (t))\Bigr ) - \theta t \delta (t) \bigl \langle Ax(t) - b, \dot{\lambda }(t)\bigr \rangle \nonumber \\&\quad + \theta t \delta (t) \bigl \langle \lambda (t) - \lambda _{*}, A\dot{x}(t)\bigr \rangle + \left\Vert \dot{x} \left( t \right) \right\Vert ^{2} + \left\Vert \dot{\lambda } \left( t \right) \right\Vert ^{2} \nonumber \\&\quad - \frac{\delta (t)}{2\ell }\left\Vert \nabla f \left( x \left( t \right) \right) - \nabla f \left( x_{*} \right) \right\Vert ^{2} - \frac{\beta \delta (t)}{2} \left\Vert Ax \left( t \right) - b \right\Vert ^{2}. \end{aligned}$$

Multiplying (B.8) by $\theta t > 0$ and then adding the result to (B.10) yields

$$\begin{aligned} \ddot{\varphi }(t) + \frac{\alpha }{t}\dot{\varphi }(t) + \theta t \dot{W}(t)&= -\bigl (\delta (t) - \theta t \dot{\delta }(t)\bigr ) \Bigl (\mathcal {L}_{\beta }\left( x(t), \lambda _{*} \right) - \mathcal {L}_{\beta }(x_{*}, \lambda (t))\Bigr ) \\&\quad -\frac{\delta (t)}{2\ell }\left\Vert \nabla f \left( x \left( t \right) \right) - \nabla f \left( x_{*} \right) \right\Vert ^{2} - \frac{\beta \delta (t)}{2}\left\Vert Ax \left( t \right) - b \right\Vert ^{2} \\&\quad +(1 - \theta \alpha ) \left\Vert \left( \dot{x} \left( t \right) , \dot{\lambda } \left( t \right) \right) \right\Vert ^{2} \\&\leqslant -\frac{\delta (t)}{2\ell }\left\Vert \nabla f \left( x \left( t \right) \right) - \nabla f \left( x_{*} \right) \right\Vert ^{2} - \frac{\beta \delta (t)}{2}\left\Vert Ax \left( t \right) - b \right\Vert ^{2}, \end{aligned}$$

where the last inequality follows from Assumption 2

$$\begin{aligned} 1 - \theta \alpha&\leqslant -\theta < 0 , \\ - \delta (t) + \theta t \dot{\delta }(t)&\leqslant (2\theta - 1)\delta (t) + \theta t \dot{\delta }(t) \leqslant 0. \end{aligned}$$

The desired result then follows after some rearranging. $\square$

### Proof of Lemma 4.5

Let $t \geqslant t_{0}$ be fixed. From (2.6) and the fact that $A^{*}\lambda _{*} = -\nabla f(x_{*})$, we have

B.11 $$\begin{aligned}&\delta ^{2} \left( t \right) \bigl \Vert \nabla f(x(t)) - \nabla f(x_{*}) + \beta A^{*}(Ax(t) - b)\bigr \Vert ^{2} \nonumber \\&\quad = \left\| \ddot{x}(t) + \frac{\alpha }{t}\dot{x}(t) + \delta (t) A^{*}\bigl (\lambda (t) - \lambda _{*} + \theta t \dot{\lambda }(t)\bigr )\right\| ^{2} \nonumber \\&\quad = \left\| \ddot{x}(t) + \frac{\alpha }{t}\dot{x}(t)\right\| ^{2} + \delta ^{2}(t) \bigl \Vert A^{*}\bigl ( \lambda (t) - \lambda _{*} + \theta t \dot{\lambda }(t)\bigr )\bigr \Vert ^{2} \nonumber \\&\qquad + 2 \delta (t) \left\langle \ddot{x}(t) + \frac{\alpha }{t}\dot{x}(t), A^{*}\bigl (\lambda (t) - \lambda _{*}\bigr )\right\rangle \nonumber \\&\qquad + 2\theta t \delta (t) \bigl \langle \ddot{x}(t), A^{*} \dot{\lambda }(t)\bigr \rangle + 2\alpha \theta t \delta (t) \bigl \langle \dot{x}(t), A^{*} \dot{\lambda }(t)\bigr \rangle . \end{aligned}$$

Again using (2.6) yields

B.12 $$\begin{aligned} \delta ^{2} \left( t \right) \left\Vert Ax \left( \left( t \right) \right) - b \right\Vert ^{2}&= \left\| \ddot{\lambda }(t) + \frac{\alpha }{t}\dot{\lambda }(t) - \theta t \delta (t) A\dot{x}(t)\right\| ^{2} \nonumber \\&= \left\| \ddot{\lambda }(t) + \frac{\alpha }{t}\dot{\lambda }(t)\right\| ^{2} + \theta ^{2} t^{2} \delta ^{2} \left( t \right) \left\Vert A \dot{x} \left( t \right) \right\Vert ^{2} \nonumber \\&\quad -2\theta t \delta (t) \bigl \langle \ddot{\lambda }(t), A\dot{x}(t)\bigr \rangle - 2\alpha \theta \delta (t) \bigl \langle \dot{\lambda }(t), A\dot{x}(t)\bigr \rangle \end{aligned}$$

Adding (B.11) and (B.12) together produces

B.13 $$\begin{aligned}&\delta ^{2} \left( t \right) \bigl \Vert \nabla f(x(t)) - \nabla f(x_{*}) + \beta A^{*}(Ax(t) - b)\bigr \Vert ^{2} + \delta ^{2} \left( t \right) \left\Vert Ax \left( \left( t \right) \right) - b \right\Vert ^{2} \nonumber \\&\quad = \left\| \bigl (\ddot{x}(t), \ddot{\lambda }(t)\bigr ) + \frac{\alpha }{t}\bigl (\dot{x}(t), \dot{\lambda }(t)\bigr )\right\| ^{2} + \delta ^{2}(t)\bigl \Vert A^{*}\bigl ( \lambda (t) - \lambda _{*}\nonumber \\&\qquad + \theta t \dot{\lambda }(t)\bigr )\bigr \Vert ^{2} + \theta ^{2} t^{2} \delta ^{2}(t) \left\Vert A \dot{x} \left( t \right) \right\Vert ^{2} \nonumber \\&\qquad + 2 \theta t \delta (t) \bigl \langle \ddot{x}(t), A^{*}\dot{\lambda }(t)\bigr \rangle - 2 \theta t \delta (t) \bigl \langle \ddot{\lambda }(t), A\dot{x}(t)\bigr \rangle \nonumber \\&\qquad + 2 \delta (t) \left\langle \ddot{x}(t) + \frac{\alpha }{t}\dot{x}(t), A^{*}(\lambda (t) - \lambda _{*})\right\rangle . \end{aligned}$$

On the one hand, we have

B.14 $$\begin{aligned}&\theta ^{2} t^{2} \delta ^{2}(t) \left\Vert A \dot{x} \left( t \right) \right\Vert ^{2} + 2\theta t \delta (t) \bigl \langle \ddot{x}(t), A^{*}\dot{\lambda }(t)\bigr \rangle - 2 \theta t \delta (t) \bigl \langle \ddot{\lambda }(t), A\dot{x}(t)\bigr \rangle \nonumber \\&\quad = \theta ^{2} t^{2} \delta ^{2}(t) \bigl \Vert \bigl (A^{*}\dot{\lambda }(t), -A\dot{x}(t)\bigr )\bigr \Vert ^{2} - \theta ^{2} t^{2} \delta ^{2}(t) \bigl \Vert A^{*} \dot{\lambda }(t)\bigr \Vert ^{2} \nonumber \\&\qquad + 2 \theta t \delta (t) \bigl \langle \bigl (\ddot{x}(t), \ddot{\lambda }(t)\bigr ), \bigl ( A^{*}\dot{\lambda }(t), - A\dot{x}(t)\bigr )\bigr \rangle \nonumber \\&\quad = - \bigl \Vert \bigl (\ddot{x}(t), \ddot{\lambda }(t)\bigr )\bigr \Vert ^{2} + \bigl \Vert \bigl (\ddot{x}(t), \ddot{\lambda }(t)\bigr ) + \theta t \delta (t) A^{*}\bigl ( A^{*}\dot{\lambda }(t), -A\dot{x}(t)\bigr )\bigr \Vert ^{2}\nonumber \\&\qquad - \theta ^{2} t^{2} \delta ^{2}(t) \bigl \Vert A^{*}\dot{\lambda }(t)\bigr \Vert ^{2} \nonumber \\&\quad \geqslant - \bigl \Vert \bigl (\ddot{x}(t), \ddot{\lambda }(t)\bigr )\bigr \Vert ^{2} - \theta ^{2} t^{2} \delta ^{2}(t) \bigl \Vert A^{*}\dot{\lambda }(t)\bigr \Vert ^{2}. \end{aligned}$$

On the other hand, it holds

B.15 $$\begin{aligned}&\left\| \bigl (\ddot{x}(t), \ddot{\lambda }(t)\bigr ) + \frac{\alpha }{t}\bigl (\dot{x}(t), \dot{\lambda }(t)\bigr )\right\| ^{2} - \bigl \Vert \bigl (\ddot{x}(t), \ddot{\lambda }(t)\bigr )\bigr \Vert ^{2} \nonumber \\&\quad = \frac{\alpha ^{2}}{t^{2}} \bigl \Vert \bigl (\dot{x}(t), \dot{\lambda }(t)\bigr )\bigr \Vert ^{2} + 2\frac{\alpha }{t}\bigl \langle \bigl (\ddot{x}(t), \ddot{\lambda }(t)\bigr ), \bigl ( \dot{x}(t), \dot{\lambda }(t)\bigr )\bigr \rangle \geqslant \frac{\alpha }{t} \frac{d}{dt}\bigl \Vert \bigl ( \dot{x}(t), \dot{\lambda }(t)\bigr )\bigr \Vert ^{2}. \end{aligned}$$

Moreover,

B.16 $$\begin{aligned}&\delta (t)\bigl \Vert A^{*}\bigl ( \lambda (t) - \lambda _{*} + \theta t \dot{\lambda }(t)\bigr )\bigr \Vert ^{2} - \theta ^{2} t^{2} \delta (t) \bigl \Vert A^{*} \dot{\lambda }(t)\bigr \Vert ^{2} \nonumber \\&\quad = \delta (t)\bigl \Vert A^{*}( \lambda (t) - \lambda _{*})\bigr \Vert ^{2} + 2\theta t \delta (t) \bigl \langle A A^{*}( \lambda (t) - \lambda _{*}), \dot{\lambda (t)}\bigr \rangle \nonumber \\&\quad = \bigl ((1 - \theta )\delta (t) - \theta t \dot{\delta }(t)\bigr ) \bigl \Vert A^{*} \bigl ( \lambda (t) - \lambda _{*}\bigr )\bigr \Vert ^{2} + \theta \delta (t) \bigl \Vert A^{*}( \lambda (t) - \lambda _{*})\bigr \Vert ^{2} \nonumber \\&\qquad + \theta t \dot{\delta }(t) \left\Vert A^{*} \left( \lambda \left( t \right) - \lambda _{*} \right) \right\Vert ^{2} + \theta \frac{d}{dt} \bigl \Vert A^{*}(\lambda (t) - \lambda _{*})\Vert ^{2} \nonumber \\&\quad = \bigl ((1 - \theta )\delta (t) - \theta t \dot{\delta }(t)\bigr ) \left\Vert A^{*} \left( \lambda \left( t \right) - \lambda _{*} \right) \right\Vert ^{2} + \theta \frac{d}{dt}\Bigl (t \delta (t) \bigl \Vert A^{*}(\lambda (t) - \lambda _{*})\bigr \Vert ^{2}\Bigr ). \end{aligned}$$

Now, using (B.14), (B.15) and (B.16) in (B.13) yields

$$\begin{aligned}&\delta ^{2} \left( t \right) \left( t \right) \bigl \Vert \nabla f(x(t)) - \nabla f(x_{*}) + \beta A^{*}(Ax(t) - b)\bigr \Vert ^{2} + \delta ^{2} \left( t \right) \left\Vert Ax \left( \left( t \right) \right) - b \right\Vert ^{2} \\&\quad \geqslant \left\| \bigl (\ddot{x}(t), \ddot{\lambda }(t)\bigr ) + \frac{\alpha }{t}\bigl (\dot{x}(t), \dot{\lambda }(t)\bigr )\right\| ^{2} + \delta ^{2}(t)\bigl \Vert A^{*}\bigl ( \lambda (t) - \lambda _{*} + \theta t \dot{\lambda }(t)\bigr )\bigr \Vert ^{2} \\&\qquad - \bigl \Vert \bigl ( \ddot{x}(t), \ddot{\lambda }(t)\bigr )\bigr \Vert ^{2} - \theta ^{2} t^{2} \delta ^{2}(t) \bigl \Vert A^{*}\dot{\lambda }(t)\bigr \Vert ^{2} + 2 \delta (t) \left\langle \ddot{x}(t) + \frac{\alpha }{t}\dot{x}(t), A^{*}(\lambda (t) - \lambda _{*})\right\rangle \\&\quad \geqslant \frac{\alpha }{t}\frac{d}{dt}\bigl \Vert \bigl (\dot{x}(t), \dot{\lambda }(t)\bigr )\bigr \Vert ^{2} + 2\delta (t) \left\langle \ddot{x}(t) + \frac{\alpha }{t}\dot{x}(t), A^{*}(\lambda (t) - \lambda _{*})\right\rangle \\&\qquad +\delta (t) \left[ \bigl ((1 - \theta )\delta (t) - \theta t \dot{\delta }(t)\bigr )\bigl \Vert A^{*}(\lambda (t) - \lambda _{*})\bigr \Vert ^{2} \right. \\&\qquad \left. + \theta \frac{d}{dt} \Bigl ( t \delta (t) \left\Vert A^{*} \left( \lambda \left( t \right) - \lambda _{*} \right) \right\Vert ^{2}\Bigr )\right] \end{aligned}$$

Finally, since

$$\begin{aligned}&\bigl \Vert \nabla f(x(t)) - \nabla f(x_{*}) + \beta A^{*}(Ax(t) - b)\bigr \Vert ^{2} + \left\Vert Ax \left( \left( t \right) \right) - b \right\Vert ^{2} \\&\quad \leqslant 2\left\Vert \nabla f \left( x \left( t \right) \right) - \nabla f \left( x_{*} \right) \right\Vert ^{2} + (2\beta \Vert A\Vert ^{2} + 1) \left\Vert Ax \left( t \right) - b \right\Vert ^{2}, \end{aligned}$$

the conclusion follows after dividing the inequality by $\delta (t)$. $\square$

### Proof of Lemma 4.6

For every $t \geqslant t_{0}$, by summing up the two inequalities produced by Lemmas 4.3 and 4.5 we deduce that

B.17 $$\begin{aligned}&\ddot{\varphi }(t) + \frac{\alpha }{t}\dot{\varphi }(t) + \theta t \dot{W}(t) + \frac{\alpha }{t\delta (t)}\bigl \Vert \bigl (\dot{x}(t), \dot{\lambda }(t)\bigr )\bigr \Vert ^{2} \nonumber \\&\quad + \theta \frac{d}{dt}\Bigl (t\delta (t) \bigl \Vert A^{*}(\lambda (t) - \lambda _{*})\bigr \Vert ^{2}\Bigr ) + 2\left\langle \ddot{x}(t) + \frac{\alpha }{t}\dot{x}(t), A^{*}(\lambda (t) - \lambda _{*})\right\rangle \nonumber \\&\qquad \leqslant -\bigl ( (1 - \theta )\delta (t) - \theta t \dot{\delta }(t)\bigr )\bigl \Vert A^{*}(\lambda (t) - \lambda _{*})\bigr \Vert ^{2} \nonumber \\&\quad + \left( 2 - \frac{1}{2\ell }\right) \delta (t) \left\Vert \nabla f \left( x \left( t \right) \right) - \nabla f \left( x_{*} \right) \right\Vert ^{2} \nonumber \\&\quad +\left( 2\beta ^{2} \Vert A\Vert ^{2} + 1 - \frac{\beta }{2}\right) \delta (t) \left\Vert Ax \left( t \right) - b \right\Vert ^{2} \nonumber \\&\qquad \leqslant -\bigl ( (1 - \theta )\delta (t) - \theta t \dot{\delta }(t)\bigr )\bigl \Vert A^{*}(\lambda (t) - \lambda _{*})\bigr \Vert ^{2} \nonumber \\&\quad + C_{3} \delta (t) \left\Vert \nabla f \left( x \left( t \right) \right) - \nabla f \left( x_{*} \right) \right\Vert ^{2} \nonumber \\&\quad +C_{4} \delta (t) \left\Vert Ax \left( t \right) - b \right\Vert ^{2}, \end{aligned}$$

where

$$\begin{aligned} C_{3}:= \left[ 2 - \frac{1}{2\ell }\right] _{+} \geqslant 0 \quad \text {and} \quad C_{4}:= \left[ 2\beta ^{2}\Vert A\Vert ^{2} + 1 - \frac{\beta }{2}\right] _{+} \geqslant 0. \end{aligned}$$

Mutiplying (B.17) by $t^{\alpha }$ and integrating from $t_{0}$ to *t*, we obtain

B.18 $$\begin{aligned}&I_{1}(t) + \theta I_{2}(t) + \alpha I_{3}(t) + \theta I_{4}(t) + 2 I_{5}(t) \nonumber \\&\quad \leqslant \int _{t_{0}}^{t}s^{\alpha } \bigl ((1 - \theta )\delta (s) - \theta s \dot{\delta }(s)\bigr ) \bigl \Vert A^{*}(\lambda (s) \nonumber \\&\qquad - \lambda _{*})\bigr \Vert ^{2} ds + C_{3} \int _{t_{0}}^{t} s^{\alpha } \delta (s) \Vert \nabla f(x(s)) - \nabla f(x_{*})\Vert ^{2} ds \nonumber \\&\qquad + C_{4}\int _{t_{0}}^{t}s^{\alpha } \delta (s) \Vert Ax(s) - b\Vert ^{2} ds, \end{aligned}$$

where

$$\begin{aligned} I_{1}(t)&:= \int _{t_{0}}^{t}\bigl (s^{\alpha } \ddot{\varphi }(s) + \alpha s^{\alpha - 1}\dot{\varphi }(s)\bigr ) ds, \\ I_{2}(t)&:= \int _{t_{0}}^{t} s^{\alpha + 1}\dot{W}(s) ds, \\ I_{3}(t)&:= \int _{t_{0}}^{t} \frac{s^{\alpha - 1}}{\delta (s)} \frac{d}{ds} \bigl \Vert \bigl (\dot{x}(s), \dot{\lambda }(s)\bigr )\bigr \Vert ^{2} ds, \\ I_{4}(t)&:= \int _{t_{0}}^{t} s^{\alpha } \frac{d}{ds}\Bigl (s\delta (s) \bigl \Vert A^{*}(\lambda (s) - \lambda _{*})\bigr \Vert ^{2}\Bigr ) ds, \\ I_{5}(t)&:= \int _{t_{0}}^{t}\bigl \langle s^{\alpha } \ddot{x}(s) + \alpha s^{\alpha - 1}\dot{x}(s), A^{*}(\lambda (s) - \lambda _{*})\bigr \rangle ds. \end{aligned}$$

We will furnish five different inequalities from computing each of these integrals separately. Let $t\geqslant t_{0}$ be fixed.

$\bullet$ *The integral* $I_{1}(t)$. By the chain rule, for $s \geqslant t_{0}$ it holds

$$\begin{aligned} s^{\alpha }\ddot{\varphi }(s) + \alpha s^{\alpha - 1}\dot{\varphi }(s) = \frac{d}{ds}\bigl ( s^{\alpha } \dot{\varphi }(s)\bigr ), \end{aligned}$$

which leads to

B.19 $$\begin{aligned} 0 = I_{1}(t) - t^{\alpha }\dot{\varphi }(t) + t_{0}^{\alpha }\dot{\varphi }(t_{0}) \leqslant I_{1}(t) - t^{\alpha }\dot{\varphi }(t) + |t_{0}^{\alpha }\dot{\varphi }(t_{0})|. \end{aligned}$$

$\bullet$ *The integrals* $I_{2}(t)$ *and*  $I_{4}(t)$. Integration by parts gives

$$\begin{aligned} I_{2}(t) + I_{4}(t)&= t^{\alpha + 1}W(t) - t_{0}^{\alpha + 1}W(t_{0}) \\&\quad - (\alpha + 1)\int _{t_{0}}^{t}s^{\alpha }W(s)ds + t^{\alpha + 1} \delta (t) \left\Vert A^{*} \left( \lambda \left( t \right) - \lambda _{*} \right) \right\Vert ^{2} \\&\quad - t_{0}^{\alpha + 1} \delta (t_{0}) \bigl \Vert A^{*}(\lambda (t_{0}) - \lambda _{*})\bigr \Vert ^{2}\\&\quad - \alpha \int _{t_{0}}^{t}s^{\alpha } \delta (s) \left\Vert A^{*}(\lambda (s) - \lambda _{*}) \right\Vert ^{2} ds , \end{aligned}$$

and from here

B.20 $$\begin{aligned}&t^{\alpha + 1} \delta (t) \left\Vert A^{*} \left( \lambda \left( t \right) - \lambda _{*} \right) \right\Vert ^{2} \leqslant t^{\alpha + 1}W(t) + t^{\alpha + 1} \delta (t) \left\Vert A^{*} \left( \lambda \left( t \right) - \lambda _{*} \right) \right\Vert ^{2} \nonumber \\&\quad = I_{2}(t) + I_{4}(t) + t_{0}^{\alpha + 1}W(t_{0}) + t_{0}^{\alpha + 1} \delta (t_{0}) \bigl \Vert A^{*}(\lambda (t_{0}) - \lambda _{*})\bigr \Vert ^{2} \nonumber \\&\qquad + (\alpha + 1)\int _{t_{0}}^{t}s^{\alpha }W(s)ds \nonumber \\&\qquad + \alpha \int _{t_{0}}^{t}s^{\alpha } \delta (s) \left\Vert A^{*}(\lambda (s) - \lambda _{*}) \right\Vert ^{2} ds . \end{aligned}$$

$\bullet$ *The integral* $I_{3}(t)$. Again by integrating by parts, we get

$$\begin{aligned} I_{3}(t)&= \frac{t^{\alpha - 1}}{\delta (t)} \bigl \Vert \bigl (\dot{x}(t), \dot{\lambda }(t)\bigr )\bigr \Vert ^{2} - \frac{t_{0}^{\alpha - 1}}{\delta (t_{0})} \bigl \Vert \bigl (\dot{x}(t_{0}), \dot{\lambda }(t_{0})\bigr )\bigr \Vert ^{2} \\&\quad - \int _{t_{0}}^{t} \left[ \frac{(\alpha - 1) s^{\alpha - 2} \delta (s) - s^{\alpha - 1}\dot{\delta }(s)}{\delta ^{2}(s)}\right] \bigl \Vert \bigl (\dot{x}(s), \dot{\lambda }(s)\bigr )\bigr \Vert ^{2} ds. \end{aligned}$$

For $s\geqslant t_{0}$, according to Assumption 2 we have $\dot{\delta }(s) \geqslant 0$, hence $\delta$ is monotonically increasing and therefore

$$\begin{aligned} \frac{(\alpha - 1) s^{\alpha - 2} \delta (s) - s^{\alpha - 1} \delta (s)}{\delta ^{2}(s)} \leqslant \frac{(\alpha - 1)s^{\alpha - 2} \delta (s)}{\delta ^{2}(s)} \leqslant \frac{(\alpha - 1)s^{\alpha }}{t_{0}^{2} \delta (t_{0})}. \end{aligned}$$

It follows that

B.21 $$\begin{aligned} 0&\leqslant \frac{t^{\alpha - 1}}{\delta (t)}\bigl \Vert \bigl (\dot{x}(t), \dot{\lambda }(t)\bigr )\bigr \Vert ^{2} \nonumber \\&= I_{3}(t) + \frac{t_{0}^{\alpha - 1}}{\delta (t_{0})}\bigl \Vert \bigl (\dot{x}(t_{0}), \dot{\lambda }(t_{0})\bigr )\bigr \Vert ^{2} \nonumber \\&\qquad + \int _{t_{0}}^{t} \left[ \frac{(\alpha - 1) s^{\alpha - 2} \delta (s) - s^{\alpha - 1}\dot{\delta }(s)}{\delta ^{2}(s)}\right] \bigl \Vert \bigl (\dot{x}(s), \dot{\lambda }(s)\bigr )\bigr \Vert ^{2} ds \nonumber \\&\leqslant I_{3}(t) + \frac{t_{0}^{\alpha - 1}}{\delta (t_{0})}\bigl \Vert \bigl (\dot{x}(t_{0}), \dot{\lambda }(t_{0})\bigr )\bigr \Vert ^{2} + \frac{\alpha - 1}{t_{0}^{2} \delta (t_{0})}\int _{t_{0}}^{t} s^{\alpha } \bigl \Vert \bigl ( \dot{x}(s), \dot{\lambda }(s)\bigr )\bigr \Vert ^{2} ds. \end{aligned}$$

$\bullet$ *The integral* $I_{5}(t)$. Integration by parts entails

$$\begin{aligned} I_{5}(t)&= \int _{t_{0}}^{t} \left\langle \frac{d}{ds}\bigl ( s^{\alpha } \dot{x}(s)\bigr ), A^{*}(\lambda (s) - \lambda _{*})\right\rangle ds \\&= t^{\alpha } \bigl \langle \dot{x}(t), A^{*}(\lambda (t) - \lambda _{*})\bigr \rangle - t_{0}^{\alpha } \bigl \langle \dot{x}(t_{0}), A^{*}(\lambda (t_{0}) - \lambda _{*})\bigr \rangle \\&\qquad - \int _{t_{0}}^{t} s^{\alpha } \bigl \langle \dot{x}(s), A^{*}\dot{\lambda }(s)\bigr \rangle ds. \end{aligned}$$

By the Cauchy–Schwarz inequality, we deduce that

$$\begin{aligned} \int _{t_{0}}^{t} s^{\alpha } \bigl \langle \dot{x}(s), A^{*}\dot{\lambda }(s)\bigr \rangle ds\leqslant & {} \left\Vert A \right\Vert \int _{t_{0}}^{t} s^{\alpha } \left\Vert \dot{x}(s) \right\Vert \left\Vert \dot{\lambda }(s) \right\Vert ds \\\leqslant & {} \dfrac{\left\Vert A \right\Vert }{2} \int _{t_{0}}^{t} s^{\alpha }\Bigl ( \bigl \Vert \dot{x}(s)\bigr \Vert ^{2} + \bigl \Vert \dot{\lambda }(s)\bigr \Vert ^{2}\Bigr ) ds, \end{aligned}$$

and thus

B.22 $$\begin{aligned} \begin{aligned} 0&\leqslant I_{5}(t) - t^{\alpha } \bigl \langle \dot{x}(t), A^{*}(\lambda (t) - \lambda _{*})\bigr \rangle + \bigl |t_{0}^{\alpha } \bigl \langle \dot{x}(t_{0}), A^{*}(\lambda (t_{0}) - \lambda _{*})\bigr \rangle \bigr | \\&\quad + \dfrac{\left\Vert A \right\Vert }{2} \int _{t_{0}}^{t} s^{\alpha } \bigl \Vert \bigl ( \dot{x}(s), \dot{\lambda }(s)\bigr )\bigr \Vert ^{2} ds. \end{aligned} \end{aligned}$$

Now, summing up the inequalities (B.19), (B.20), (B.21), and (B.22), then we proceed to employ (B.18) and obtain

$$\begin{aligned}&\theta t^{\alpha + 1} \delta (t) \left\Vert A^{*} \left( \lambda \left( t \right) - \lambda _{*} \right) \right\Vert ^{2} \\&\quad \leqslant I_{1}(t) + \theta I_{2}(t) + \alpha I_{3}(t) + \theta I_{4}(t) + 2I_{5} - t^{\alpha } \dot{\varphi }(t) \\&\qquad {+\int _{t_{0}}^{t}s^{\alpha } \left[ \theta (\alpha + 1) W(s) + \left( \frac{\alpha (\alpha - 1)}{t_{0}^{2} \delta (t_{0})} + \left\Vert A \right\Vert \right) \bigl \Vert \bigl (\dot{x}(s), \dot{\lambda }(s)\bigr )\bigr \Vert ^{2}\right] ds} \\&\qquad + \theta \alpha \int _{t_{0}}^{t} s^{\alpha } \delta (s) \left\Vert A^{*}(\lambda (s) - \lambda _{*}) \right\Vert ^{2} ds - 2 t^{\alpha } \bigl \langle \dot{x}(t), A^{*}(\lambda (t) - \lambda _{*})\bigr \rangle + C_{5} \\&\quad \leqslant -t^{\alpha } \dot{\varphi }(t) + \int _{t_{0}}^{t} s^{\alpha } V(s) ds + \int _{t_{0}}^{t} s^{\alpha } \Bigl [ \bigl (\theta (\alpha + 1) - 1\bigr )\delta (t) + \theta s \dot{\delta }(s)\Bigr ] \\&\qquad \left\Vert A^{*}(\lambda (s) - \lambda _{*}) \right\Vert ^{2} ds \\&\qquad -2 t^{\alpha } \bigl \langle \dot{x}(t), A^{*}(\lambda (t) - \lambda _{*})\bigr \rangle + C_{5}, \end{aligned}$$

where recall that *V* was given by

$$\begin{aligned} V(s)&:= \theta (\alpha + 1) W(s) + \left( \frac{\alpha (\alpha - 1)}{t_{0}^{2} \delta (_{0})} + \left\Vert A \right\Vert \right) \bigl \Vert \bigl ( \dot{x}(s), \dot{\lambda }(s)\bigr )\bigr \Vert ^{2} \\&\quad + C_{3} \delta (s) \Vert \nabla f(x(s)) - \nabla f(x_{*})\Vert ^{2} + C_{4} \delta (s) \Vert Ax(s) - b\Vert ^{2}, \end{aligned}$$

and the constant $C_{5}$ is given by

$$\begin{aligned} C_{5}&:= t_{0}^{\alpha } |\dot{\varphi }(t_{0})| + \theta t_{0}^{\alpha + 1}W(t_{0}) + \alpha \frac{t_{0}^{\alpha - 1}}{\delta (t_{0})}\bigl \Vert \bigl (\dot{x}(t_{0}, \dot{\lambda }(t_{0}))\bigr )\bigr \Vert ^{2} \\&\quad +\theta t_{0}^{\alpha + 1} \delta (t_{0}) \bigl \Vert A^{*}(\lambda (t_{0}) - \lambda _{*})\bigr \Vert ^{2} + 2 t_{0}^{\alpha } \bigl | \bigl \langle \dot{x}(t_{0}), A^{*}(\lambda (t_{0}) - \lambda _{*})\bigr \rangle \bigr | \geqslant 0. \end{aligned}$$

We come then to the desired result.
